# ABCA6 affects the malignancy of Ewing sarcoma cells via cholesterol-guided inhibition of the IGF1R/AKT/MDM2 axis

**DOI:** 10.1007/s13402-022-00713-5

**Published:** 2022-09-23

**Authors:** Michela Pasello, Anna Maria Giudice, Camilla Cristalli, Maria Cristina Manara, Caterina Mancarella, Alessandro Parra, Massimo Serra, Giovanna Magagnoli, Florencia Cidre-Aranaz, Thomas G. P. Grünewald, Carla Bini, Pier-Luigi Lollini, Alessandra Longhi, Davide Maria Donati, Katia Scotlandi

**Affiliations:** 1 Experimental Oncology Laboratory, IRCCS Istituto Ortopedico Rizzoli, Bologna, 40136 Italy; 2 Alma Mater Institute On Healthy Planet - Alma Healthy Planet, University of Bologna, Bologna, Italy; 3 Department of Experimental, Diagnostic and Specialty Medicine (DIMES), University of Bologna, Bologna, Italy; 4 Department of Pathology, IRCCS Istituto Ortopedico Rizzoli, Bologna, Italy; 5 Division of Translational Pediatric Sarcoma Research, German Cancer Research Center (DKFZ), German Cancer Consortium (DKTK), Heidelberg, Germany; 6 Hopp-Children’s Cancer Center (KiTZ), Heidelberg, Germany; 7 Institute of Pathology, Heidelberg University Hospital, Heidelberg, Germany; 8 Laboratory of Forensic Genetics, Department of Medical and Surgical Sciences, University of Bologna, Bologna, Italy; 9 Osteoncologia, Sarcomi dell’osso e dei Tessuti Molli e Terapie Innovative, IRCCS Istituto Ortopedico Rizzoli, Bologna, Italy; 10 Unit of 3rd Orthopaedic and Traumatologic Clinic Prevalently Oncologic, IRCCS Istituto Ortopedico Rizzoli, Bologna, Italy; 11 Department of Biomedical and Neuromotor Sciences (DIBINEM), University of Bologna, Bologna, Italy

**Keywords:** Ewing sarcoma, ABC family of transporters, Cholesterol, Statins, Tumor biomarkers

## Abstract

**Purpose:**

The relevance of the subfamily A members of ATP-binding cassette (ABCA) transporters as biomarkers of risk and response is emerging in different tumors, but their mechanisms of action have only been partially defined. In this work, we investigated their role in Ewing sarcoma (EWS), a pediatric cancer with unmet clinical issues.

**Methods:**

The expression of ABC members was evaluated by RT-qPCR in patients with localized EWS. The correlation with clinical outcome was established in different datasets using univariate and multivariate statistical methods. Functional studies were conducted in cell lines from patient-derived xenografts (PDXs) using gain- or loss-of-function approaches. The impact of intracellular cholesterol levels and cholesterol lowering drugs on malignant parameters was considered.

**Results:**

We found that *ABCA6*, which is usually poorly expressed in EWS, when upregulated became a prognostic factor of a favorable outcome in patients. Mechanistically, high expression of ABCA6 impaired cell migration and increased cell chemosensitivity by diminishing the intracellular levels of cholesterol and by constitutive IGF1R/AKT/mTOR expression/activation. Accordingly, while exposure of cells to exogenous cholesterol increased AKT/mTOR activation, the cholesterol lowering drug simvastatin inhibited IGF1R/AKT/mTOR signaling and prevented Ser166 phosphorylation of MDM2. This, in turn, favored p53 activation and enhanced pro-apoptotic effects of doxorubicin.

**Conclusions:**

Our study reveals that ABCA6 acts as tumor suppressor in EWS cells via cholesterol-mediated inhibition of IGF1R/AKT/MDM2 signaling, which promotes the pro-apoptotic effects of doxorubicin and reduces cell migration. Our findings also support a role of *ABCA6* as biomarker of EWS progression and sustains its assessment for a more rational use of statins as adjuvant drugs.

**Supplementary Information:**

The online version contains supplementary material available at 10.1007/s13402-022-00713-5.

## Introduction

Mechanisms of chemoresistance in cancer are manifold and only partially defined. They include, but are not limited to, enhanced drug efflux-pump activity, changes in the intracellular metabolic machinery, upregulation of DNA repair mechanisms, induction of growth signaling and impairment of apoptosis [1]. Recent evidence indicates a more complex role for several ATP-binding cassette (ABC) transporters in tumor progression. Some ABC transporter members, particularly ABCB1 (P-glycoprotein/MDR1), ABCC1 (Multidrug Resistance-associated Protein 1/MRP1) and ABCG2 (Breast Cancer Resistance Protein/BCRP) act as cell membrane pumps that are capable of extruding drugs from cancer cells, and their contribution to multidrug resistance is widely recognized in different tumors (for a review, see [2]). Other members have been found to play roles in the regulation of cancer cell proliferation, differentiation, migration and invasion, to mediate intracellular peptide and lipid transport (for a review, see [3, 4]) and to be part of the signaling networks that orchestrate the activation and polarization of macrophages and to affect the fate of myeloid progenitors [5, 6]. In this study, we focus on studying the impact of unconventional ABC transporters in Ewing sarcoma (EWS) and we use patient-derived xenograft (PDX) models to identify mechanisms of action for the *ABCA6* transporter that has emerged as a prognostic biomarker in EWS patients.

The ABCA subfamily of transporters consists of 12 members, whose best-defined physiological functions are related to the maintenance of lipid homeostasis and the regulation of cellular lipid transport and trafficking, including efflux from cells of cholesterol and phospholipids (*i.e.,* phosphatidylcholine, phosphatidylserine and sphingomyelin) (for a review, see [7]). These lipids are reported to severely impact many biological processes related to cancer by regulating plasma membrane cell fluidity and functionality (for a review, see [8, 9]), but information on this subfamily of transporters in cancer is dispersed, controversial and mostly limited to the ABCA1 member [8, 10]. In our current study, we found that high tumor levels of *ABCA6* were predictive of a favorable prognosis in EWS patients. We chose to study EWS, the second most frequent primary tumor of bone in the pediatric population because (i) patients still face the disadvantage of uniform, non-individualized chemotherapy, which severely impacts their quality of life and/or prognosis [11], (ii) patients who fail to respond to first-line treatments or already had metastases at diagnosis still have a dismal prognosis (overall survival rate < 40%), largely because their tumors are resistant to conventional chemotherapy [12, 13] and (iii) data for ABCB1, ABCG2 and ABCC1 are scarce and controversial in EWS [14–17], supporting the need of extended analysis and evaluation of novel candidates.

Our findings highlight the importance of ABCA6 as a biomarker of risk and response in EWS and provide a mechanistic explanation for its involvement in the regulation of cancer aggressiveness. High expression of this transporter impaired cell migration and increased cell chemosensitivity to DNA-damaging agents by diminishing intracellular cholesterol content, thereby decreasing the functional activity of the IGF1R/AKT/mTOR/MDM2 axis. The cholesterol-lowering drug simvastatin recapitulated similar effects and exhibited synergistic anti-proliferative and pro-apoptotic effects when combined with doxorubicin. This effect was particularly relevant in the most aggressive cells characterized by a low expression of ABCA6 and a high intracellular level of cholesterol.

## Materials and methods

### Patients selection

Patients with localized EWS who were enrolled in prospective neoadjuvant studies [18, 19] and treated at the Rizzoli Institute were included in this analysis. Based on biobank availability and a tumor tissue quality control check, 103 primary tumors were studied (25 samples: training set and 78 samples: validation set). The clinicopathological features of the patients are reported in Supplementary Table 1. Local treatment, performed after induction chemotherapy, consisted of radiation therapy, surgery or surgery followed by radiation therapy. In patients locally treated by surgery, the histological response to chemotherapy was evaluated according to the method proposed by Picci et al. [20].

For the training set, the median follow-up was 72 months (range 10–328 months). The cohort was composed of 9 patients (36%) who remained continuously free of disease (NED) and 16 patients (64%) who relapsed (REL). For the validation set, the median follow-up was 61.5 months (range 4–328 months), 44 patients (56.4%) remained continuously free of disease, and 34 (43.6%) relapsed. Clinical and follow-up data were updated to June 2020. Relapse-free survival (RFS) was calculated from the date of initial diagnosis. The clinical endpoint was the occurrence of adverse events (defined as recurrence or metastases at any site for RFS or cancer-related death for overall survival, OS).

Additionally, microarray data of 166 primary EWS tumors downloaded from the National Center for Biotechnology Information (NCBI) Gene Expression Omnibus (GEO) were analyzed. More details are provided in the Supplementary Methods.

### Preclinical studies

Functional studies were conducted on 4 cell lines derived from patient-derived xenografts (PDXs). PDX-derived cell lines, named PDX-EW#2-C, PDX-EW#3-C, PDX-EW#4-C and PDX-EW#5-C, were obtained from the respective EWS PDXs after their first passage in mice [21]. All cell lines were immediately amplified to construct liquid nitrogen stocks and were never passaged for more than 1 month upon thawing. Cells were maintained in Iscove's modified Dulbecco's medium (IMDM; Euroclone) supplemented with 10% heat-inactivated fetal bovine serum (FBS; Euroclone), penicillin (20 U/ml) and streptomycin (100 μg/ml; Sigma) in a 37 °C humidified environment at 5% CO_2_. All cell lines were authenticated by short tandem repeat PCR analysis (17 STRs analyzed; last control July 2018; POWERPLEX ESX 17 Fast System, Promega) and found to be mycoplasma-free using a MycoAlert mycoplasma detection kit (Lonza; control every 3 months).

For forced expression or silencing of ABCA6, 1 × 10^6^ PDX-EW#2-C or PDX-EW#5-C cells/well, respectively, were seeded in 6-well plates coated with fibronectin (3 μg/cm^2^; Sigma). For forced expression, cells were transfected 24 h after seeding with expression vector pCMV6-AC-GFP containing full-length *hABCA6* (Origene), and non-transfected (NT) or empty vector transfected (EV) cells were used as controls. For silencing, cells were transfected with a lentiviral pLKO.1 expression vector containing short hairpin RNAs (shRNAs) against human *ABCA6* (shABCA6). Five constructs were mixed to ensure adequate coverage of the target gene (sequences 5’-3’ of shRNA were as follows: shABCA6-1 ATTCCTGCTGTTAATTTCTGC, shABCA6-2 TTTAACTTTAAGAAACGGAGC, shABCA6-3 AATAAAGGAGAATAATGGCGC, shABCA6-4 TAGCAAAGTCTGAAAGTAGGG, shABCA6-5 TTTACCAGAAACTATGATAGC; human TRC shRNA library TRC-Hs1.0 Human; Dharmacon). Non-transfected (NT) cells or cells transfected with a pLKO.1 expression vector containing shRNA against enhanced green fluorescent protein (shGFP) were used as controls. Transfections were performed using TransIT-X2 (Mirus) according to the manufacturer’s protocols. The expression levels of ABCA6 were determined by western blot analysis after 48 h of transfection.

Anchorage-independent growth was determined in 0.33% agarose (Lonza) with a 0.5% agarose underlay. Cell motility and chemotaxis assays were performed using Transwell chambers (Costar) and scratch wound-healing assays. In vitro drug sensitivity was assessed using a 3-(4,5-dimethylthiazol-2-yl)-2,5-diphenyltetrazolium bromide (MTT) assay (TACS MTT Cell Proliferation Assays; Trevigen) according to the manufacturer’s protocol or by Trypan blue vital cell count (Sigma). Changes in mitochondrial membrane potential were assessed by flow cytometry measuring 1,1′,3,3′-tetraethylbenzimidazolcarbocyanine iodide (JC-1; Sigma) red and green fluorescence intensities. Intracellular cholesterol was detected using filipin III staining. Intracellular and supernatant cholesterol was quantified using a colorimetric Total Cholesterol Assay Kit (Cell Biolabs). Lipid extracts were obtained from 1 × 10^6^ cells using 200 μl of a chloroform:isopropanol: NP-40 (7:11:0.1, v:v:v) mixture and further processed according to the manufacturers’ protocol.

For western blot analysis, cells were lysed with phospho-protein extraction buffer supplemented with protease-phosphatase cocktail inhibitor (Sigma). Proteins of interest were detected by specific antibodies.

More details on the preclinical studies are provided in the Supplementary Methods.

### Statistical analysis

Associations between ABC transporter expression and RFS or OS were estimated by Cox proportional hazards regression analysis. RFS and OS were plotted using the Kaplan–Meier method, while the log-rank test was used to calculate univariate statistical significance of observed differences. Survivors or patients who were lost at follow-up were censored at the last contact date. All factors significantly associated with RFS in univariate analysis were entered into a Cox proportional hazards model multivariate analysis. Values of 95% confidence intervals (CIs) of hazard ratios (HRs) were provided [22]. All experiments were performed at least in triplicate, and all values are reported as the mean ± SEM. Differences among means were analyzed using unpaired two-sided Student’s t-test. Experimental data including more than 2 groups were analyzed using one-way or two-way ANOVA. Fisher’s exact test was used for association data. IC50 values were calculated from linear transformation of dose–response curves using CalcuSyn software (Biosoft). To define drug-drug interactions, the combination index (CI) was calculated with an isobologram equation using CalcuSyn software to identify synergistic (CI < 0.9), additive (0.9 ≤ CI ≤ 1.1), or antagonistic (CI > 1.1) effects according to Chou et al. [23]. All *p* values were two-sided and a *p* value < 0.05 was considered statistically significant. Statistical analyses were performed using SPSS software, version 22.0 and GraphPad Prism 6 (GraphPad Prism).

## Results

### High expression of *ABCA6* in primary tumors predicts favorable outcomes in EWS patients

To identify ABC transporters whose expression is associated with differential patient outcome in EWS, we performed an explorative quantitative reverse transcription PCR (RT-qPCR) analysis of 15 ABC transporters that were reported to play a role in drug resistance/tumor aggressiveness (for a review see [24]). The median value for each gene (Supplementary Table 2) was used as the cutoff value to stratify patients and define two categories of high or low expressors.

**Fig. 1:**
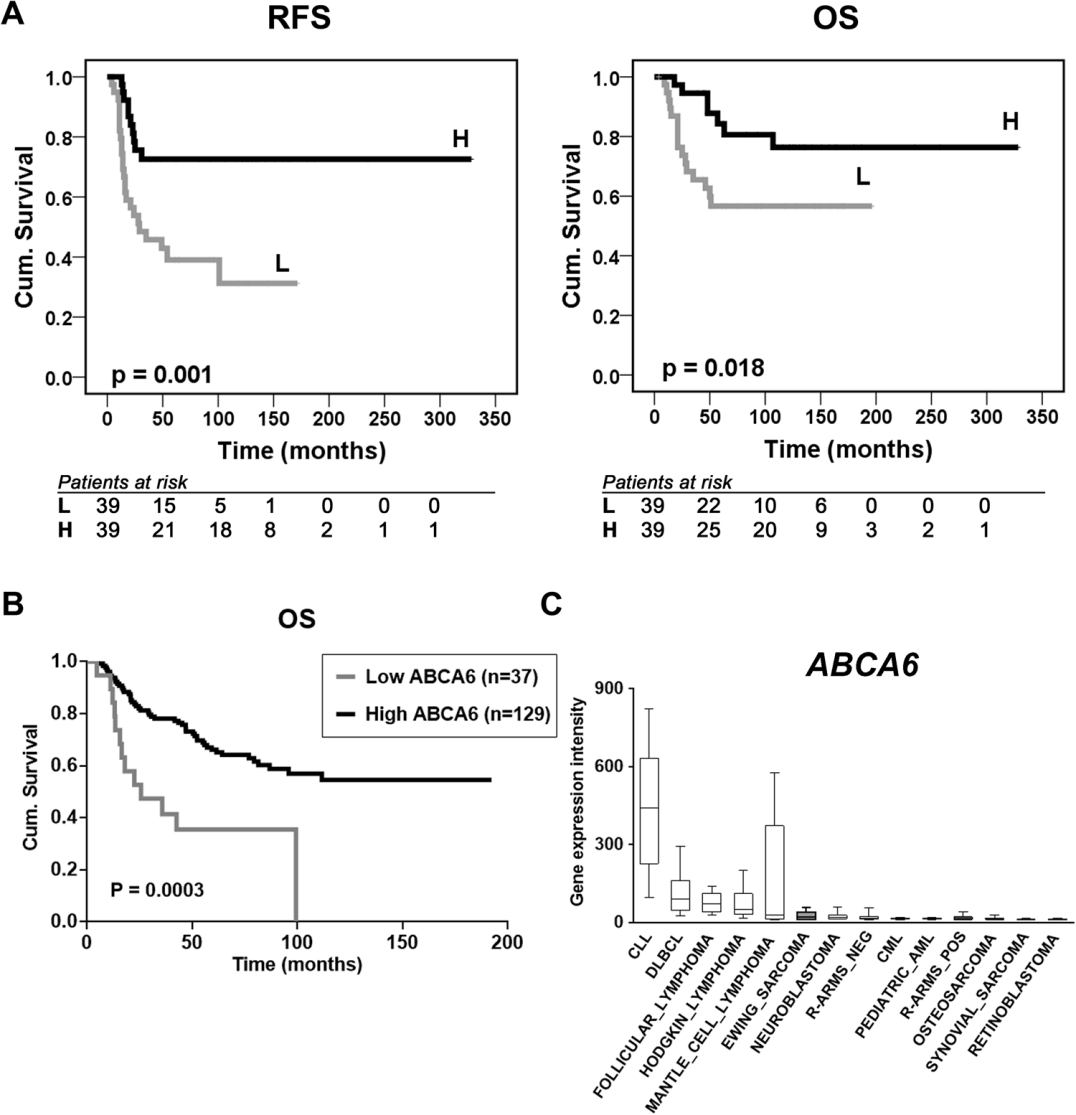
Prognostic value of *ABCA6* in primary EWS patients. **a**, Prognostic impact of *ABCA6* expression according to Kaplan–Meier curves and log-rank test in 78 EWS cases analyzed by RT-qPCR. Samples with high (H) and low (L) expression were defined according to the median value. Relapse-free survival (RFS) and overall survival (OS) were evaluated. The time scale refers to months from diagnosis. The number of patients at risk in the H and L groups is listed below each time interval. **b**, Kaplan–Meier analysis of OS of EWS patients (n = 166), stratified in two groups according to their *ABCA6* expression status in ‘high’ and ‘low’ (cut-off 22^nd^ expression percentile). All EWS tumors were profiled on Affymetrix gene expression arrays. Mantel–Haenszel test. **c**, Comparative gene expression analysis of *ABCA6* in EWS (grey color; n = 50) and other solid and hematologic (pediatric) cancers (n ≥ 20 per canter type). Gene expression data were generated on Affymetrix HG-U133Plus2.0 arrays, downloaded from public repositories, manually curated and jointly normalized using RMA and brainarray CDF. Data are displayed as box-plots: the horizontal bars represent median expression values, boxes the interquartile range, and whiskers the 10^th^–90^th^ expression percentile. CLL, chronic lymphoblastic leukemia; CML, chronic myeloid leukemia; DLBCL, diffuse large B-cell lymphoma; R-ARMS pos, alveolar rhabdomyosarcoma fusion-positive; R-ARMS neg, alveolar rhabdomyosarcoma fusion-negative; pediatric AML, pediatric acute myeloid leukemia

In the training set (25 samples from patients with primary localized EWS), the log-rank univariate analysis (Mantel-Cox test) indicated a statistically significant association for the expression of *ABCA6* and *ABCA7* with different RFS (Table 1 and Supplementary Fig. 1). However, this correlation was confirmed only for *ABCA6* in the validation set (78 samples from patients with primary localized EWS): high expression of *ABCA6* was found to be associated with favorable patient’s outcomes, using RFS and OS as a primary endpoint (Fig. 1a, Supplementary Table 3 and Supplementary Table 4). In patients with high expression of *ABCA6*, adverse events occurred in 10 out of 34 (29.4%), while in patients with low expression of *ABCA6* adverse events occurred in 24 out of 34 (70.6%) (*p* = 0.003, Fisher’s exact test). Accordingly, tumor-related death occurred in 30.4% (7 out of 23) of patients with high expression of *ABCA6,* but in 69.6% (16 out of 23) of patients with low expression of the transporter (*p* = 0.046, Fisher’s exact test). Multivariate analysis supported the statistical significance of the low expression level of *ABCA6* as an independent risk factor for poor outcomes (HR = 2.812; 95% CI = 1.226–6.445;* p* = 0.015) (Supplementary Table 5).

**Table 1:** Prognostic impact of ABC transporters in 25 patients with Ewing sarcoma. Associations with prognosis were calculated by univariate analysis using the log-rank (Mantel-Cox) test

		Relapse-Free Survival^a^		Overall Survival^b^	
Gene	*n*	Events (% RFS)	*p*-Univariate	Events (% OS)	*p*-Univariate
*ABCA2*			0.067		0.103
Low	13	10 (17.9%)		8 (34.6%)	
High	12	6 (50.0%)		4 (66.7%)	
*ABCA6*			**0.026**		0.085
Low	12	9 (20.8%)		7 (38.1%)	
High	13	7 (46.2%)		5 (61.5%)	
*ABCA7*			**0.035**		**0.023**
Low	13	11 (8.8%)		9 (26.0%)	
High	12	5 (58.3%)		3 (75.0%)	
*ABCB1*			0.530		0.215
Low	12	8 (29.2%)		7 (37.5%)	
High	13	8 (38.5%)		5 (61.5%)	
*ABCB10*			0.951		0.563
Low	12	8 (28.1%)		5 (55.6%)	
High	13	8 (38.5%)		7 (46.2%)	
*ABCC1*			0.468		0.965
Low	12	9 (18.7%)		6 (46.3%)	
High	13	7 (46.2%)		6 (53.8%)	
*ABCC2*			0.967		0.859
Low	12	8 (28.1%)		6 (46.3%)	
High	13	8 (38.5%)		6 (53.8%)	
*ABCC4*			0.055		0.055
Low	12	10 (9.7%)		8 (28.1%)	
High	13	6 (53.8%)		4 (69.2%)	
*ABCC5*			0.080		0.361
Low	12	10 (9.5%)		7 (37.5%)	
High	13	6 (53.8%)		5 (61.5%)	
*ABCC11*			0.172		0.535
Low	12	6 (46.9%)		5 (55.6%)	
High	13	10 (23.1%)		7 (46.2%)	
*ABCE1*			0.142		0.446
Low	13	10 (17.6%)		7 (42.7%)	
High	12	6 (50.0%)		5 (58.3%)	
*ABCF1*			0.065		0.348
Low	12	10 (9.5%)		7 (37.5%)	
High	13	6 (53.8%)		5 (61.5%)	
*ABCF2*			0.329		0.837
Low	12	9 (19.0%)		6 (46.3%)	
High	13	7 (46.2%)		6 (53.8%)	
*ABCF3*			0.123		0.212
Low	12	9 (19.4%)		7 (38.1%)	
High	13	7 (46.2%)		5 (61.5%)	
*ABCG2*			0.284		0.498
Low	12	8 (30.0%)		6 (47.6%)	
High	13	8 (38.5%)		6 (53.8%)	

Results in bold are significant at *p *< 0.05. ^a^RFS, relapse-free survival (median follow-up: 21 months; range 4–328 months); ^b^OS, overall survival (median follow-up: 72 months; range 10–328 months)

To validate the data obtained from our clinical samples and to limit the impact of possible technical biases, gene expression data obtained from publicly available microarray records of 166 primary EWS tumors were analyzed. The 22^nd^ percentile, which indicates a low expression of the molecule, was chosen as the optimal cutoff by a specific algorithm, which tests all possible cutoffs between the 20^th^ and 80^th^ percentile of the entire range of all gene expression values for the given gene in the given dataset. The analysis still confirmed the significant association between high expression of *ABCA6* and a better prognosis (Fig. 1b), further supporting the idea that *ABCA6* could contribute to determine the outcome for EWS patients. Additionally, publicly available data showed that *ABCA6* was expressed at lower levels in EWS compared with other pediatric tumors, particularly lymphoma/leukemia (Fig. 1c), suggesting the lack of this transporter as a peculiar feature of EWS.

### ABCA6 levels influence EWS cell migration and chemosensitivity by affecting intracellular cholesterol content

**Fig. 2:**
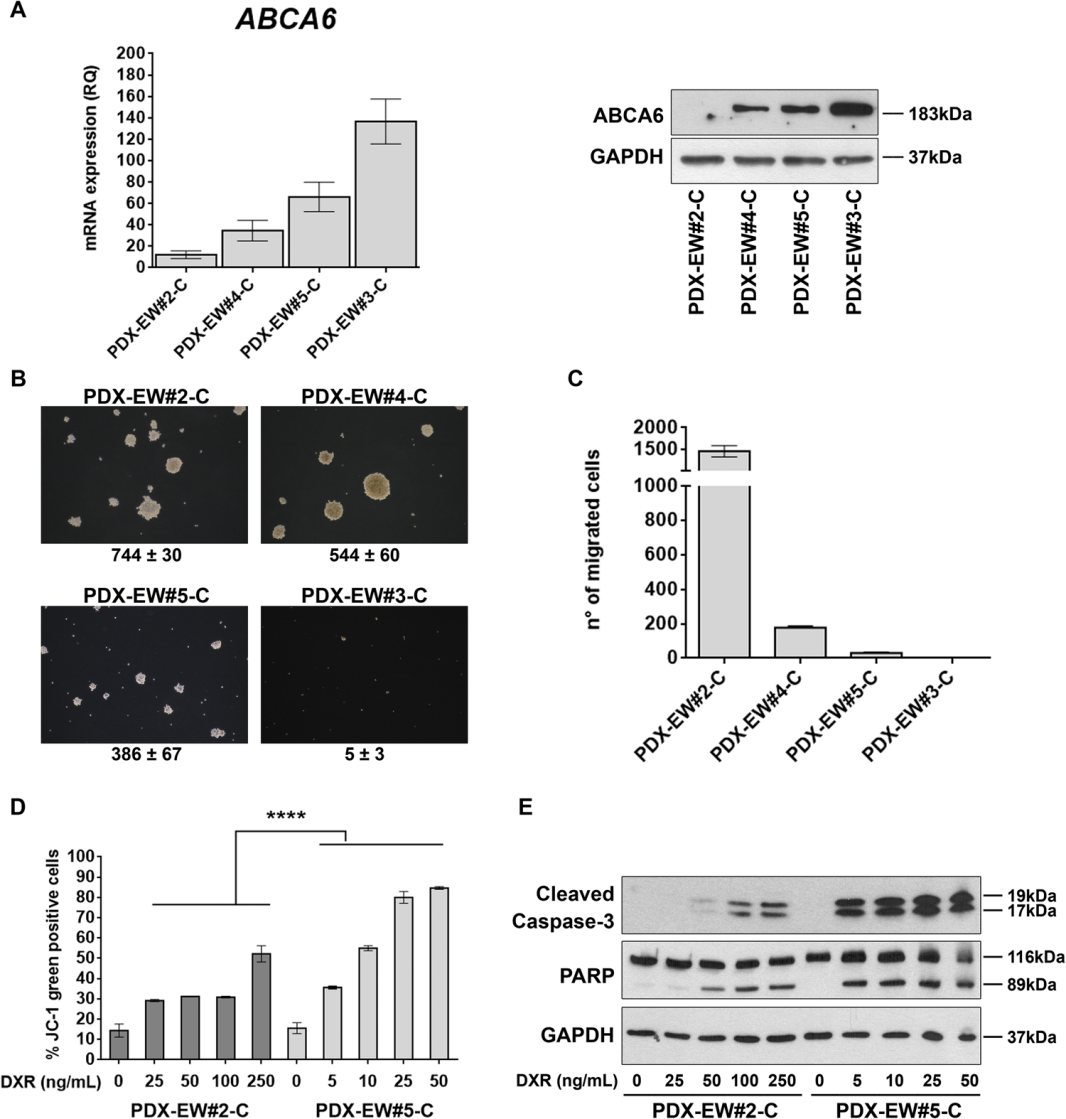
Expression of ABCA6 influences malignancy and chemosensitivity of EWS cells. **a,** *ABCA6* mRNA relative expression by RT-qPCR. Data are the mean ± SEM (*n* = 3) (left). Protein expression of ABCA6 by western blotting. GAPDH was used as a loading control (right). **b,** Colonies formation in anchorage-independent conditions of PDX-EW#5-C and PDX-EW#3-C (ABCA6^high^) and PDX-EW#2-C and PDX-EW#4-C (ABCA6^low^). Pictures are from a representative experiment (40X magnification). Data are the mean ± SEM (*n* = 3). **c**, Migratory abilities of PDX-EW#5-C and PDX-EW#3-C (ABCA6^high^) and PDX-EW#2-C and PDX-EW#4-C (ABCA6^low^). Data are the mean ± SEM (*n* = 6). **d**, Mitochondrial depolarization after cell exposure to doxorubicin (DXR, 24 h) detected by flow cytometry. A dose-dependent increase in mitochondrial depolarization was observed. Data are the mean ± SEM (*n* = 2). Drug effects were significantly higher in PDX-EW#5-C cells (ABCA6^high^) than in the PDX-EW#2-C cells (ABCA6^low^) (****p < 0.0001, two-way ANOVA). Efficacy of doxorubicin in each cell line compared to respective control was tested by one-way ANOVA, reporting significant *p* values (range from *p* < 0.05 to *p* > 0.0001). **e**, Protein expression of cleaved caspase 3 and PARP after cell exposure to DXR (24 h) by western blotting. GAPDH was used as a loading control

**Fig. 3:**
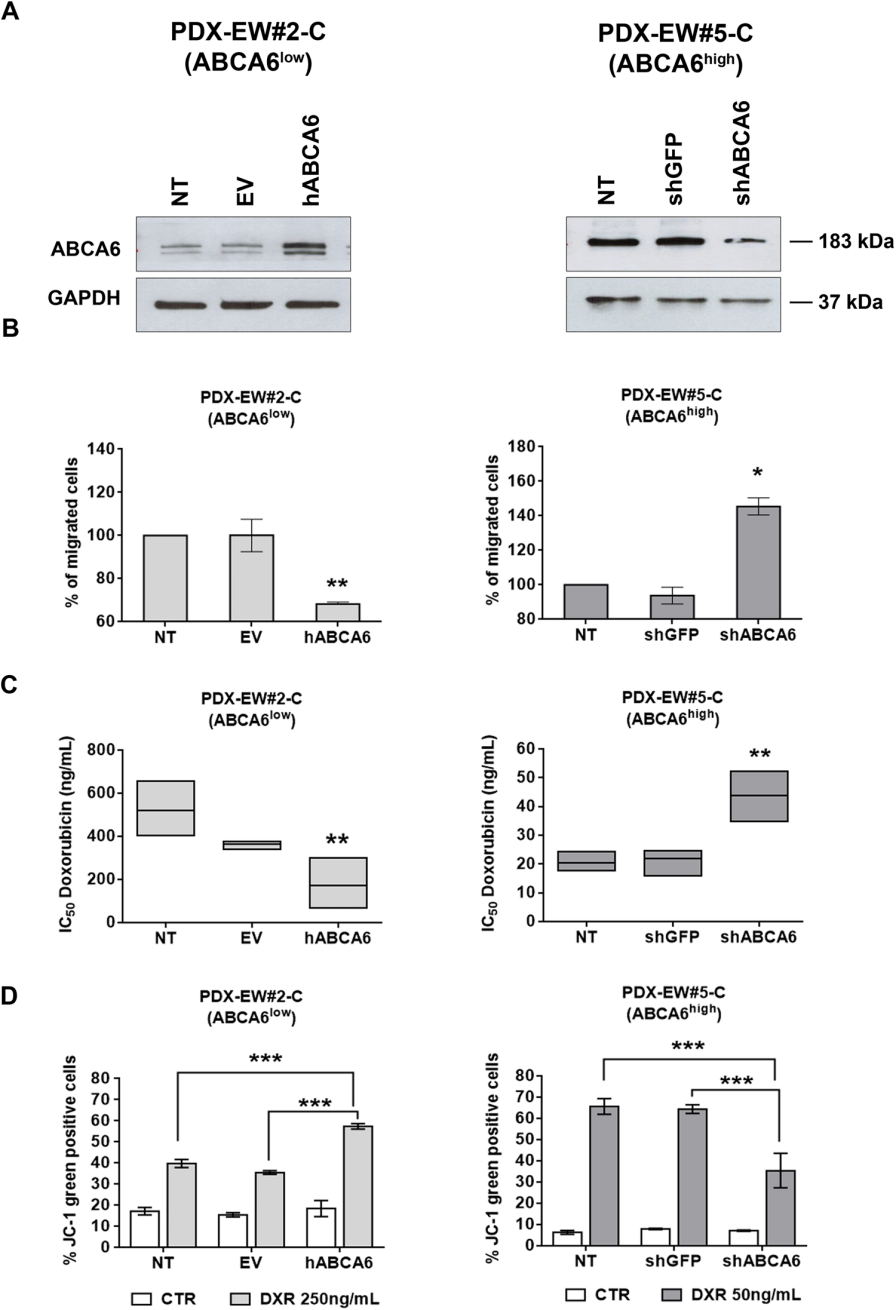
Forced expression or silencing of ABCA6 affects cell migration and chemosensitivity to doxorubicin. **a,** Expression of ABCA6 in PDX-EW#2-C cells after forced expression (left) or in PDX-EW#5-C cells after silencing (right) determined by western blotting. A representative experiment of three is shown. GAPDH was used as a loading control. **b**, Migratory ability of PDX-EW#2-C and PDX-EW#5-C transfected cells. Data are the mean ± SEM (*n* = 3); *p < 0.05, **p < 0.01, one–way ANOVA *vs* control (non-transfected cells; NT). **c**, Sensitivity to doxorubicin (DXR) of transfected cells, expressed as IC50 values, after 24 h of treatment. Data are the mean ± SEM (*n* = 3); **p < 0.01 one–way ANOVA *vs* control (non-transfected cells; NT). **d**, Mitochondrial depolarization after cells exposure to DXR (24 h) detected by flow cytometry. Sensitivity to DXR of control cells was compared to that of ABCA6 overexpressing (hABCA6) or ABCA6 silenced cells (shABCA6). Data are the mean ± SEM (*n* = 3). ***p < 0.001, two-way ANOVA

Since the expression levels of *ABCA6* in PDXs and in the corresponding PDX-derived cell lines were found to be more similar to those found in clinical samples than in conventional cell lines with a long history in culture (Supplementary Fig. 2), we decided to use the PDX-derived cell lines to study in more detail the impact that this transporter has on the regulation of tumor cell growth and migration. Among the four PDX-derived cell lines, PDX-EW#2-C cells barely expressed the transporter and PDX-EW#4-C cells showed a low expression, while PDX-EW#3-C and PDX-EW#5-C expressed ABCA6 at high levels (Fig. 2a). In keeping with clinical data, the two cell lines with a high expression of ABCA6 displayed decreased capabilities to grow in anchorage-independent conditions and to migrate (Fig. 2b and c). Due to the severe limitations on cell growth and migration that characterize the PDX-EWS#3-C cells, we decided to perform further studies on the PDX-EWS#5-C cell line that still represents the high ABCA6 condition. We found that PDX-EW#5-C cells (ABCA6^high^) exhibited a significantly enhanced sensitivity to DNA damaging chemotherapeutics doxorubicin (*p* = 0.003, Student’s t-test), etoposide (*p* = 0.004, Student’s t-test) and ifosfamide (*p* = 0.028, Student’s t-test) compared to PDX-EW#2-C cells (ABCA6^low^) (Supplementary Table 6). The exposure of cells to doxorubicin induced a dose-dependent increase in mitochondrial depolarization, which was significantly higher in PDX-EW#5-C cells (ABCA6^high^) than in PDX-EW#2-C cells (ABCA6^low^) (*p* < 0.0001, two-way ANOVA; Fig. 2d), leading to increased activation of caspase-3 and PARP cleavage (Fig. 2e). Gain- or loss-of-function approaches in PDX-EW#2-C cells (ABCA6^low^) (Fig. 3, left panel) or PDX-EW#5-C cells (ABCA6^high^), respectively (Fig. 3, right panel), confirmed that the migration of EWS cells was indeed impaired when the transporter was overexpressed (Fig. 3b, left panel) and promoted when it was silenced (Fig. 3b, right panel). Overexpression of ABCA6 also enhanced cell chemosensitivity to doxorubicin (Fig. 3c, left panel) by improving its pro-apoptotic effects (Fig. 3d, left panel), while the abrogation of its expression led to opposite results (Fig. 3c and d, right panel).

**Fig. 4:**
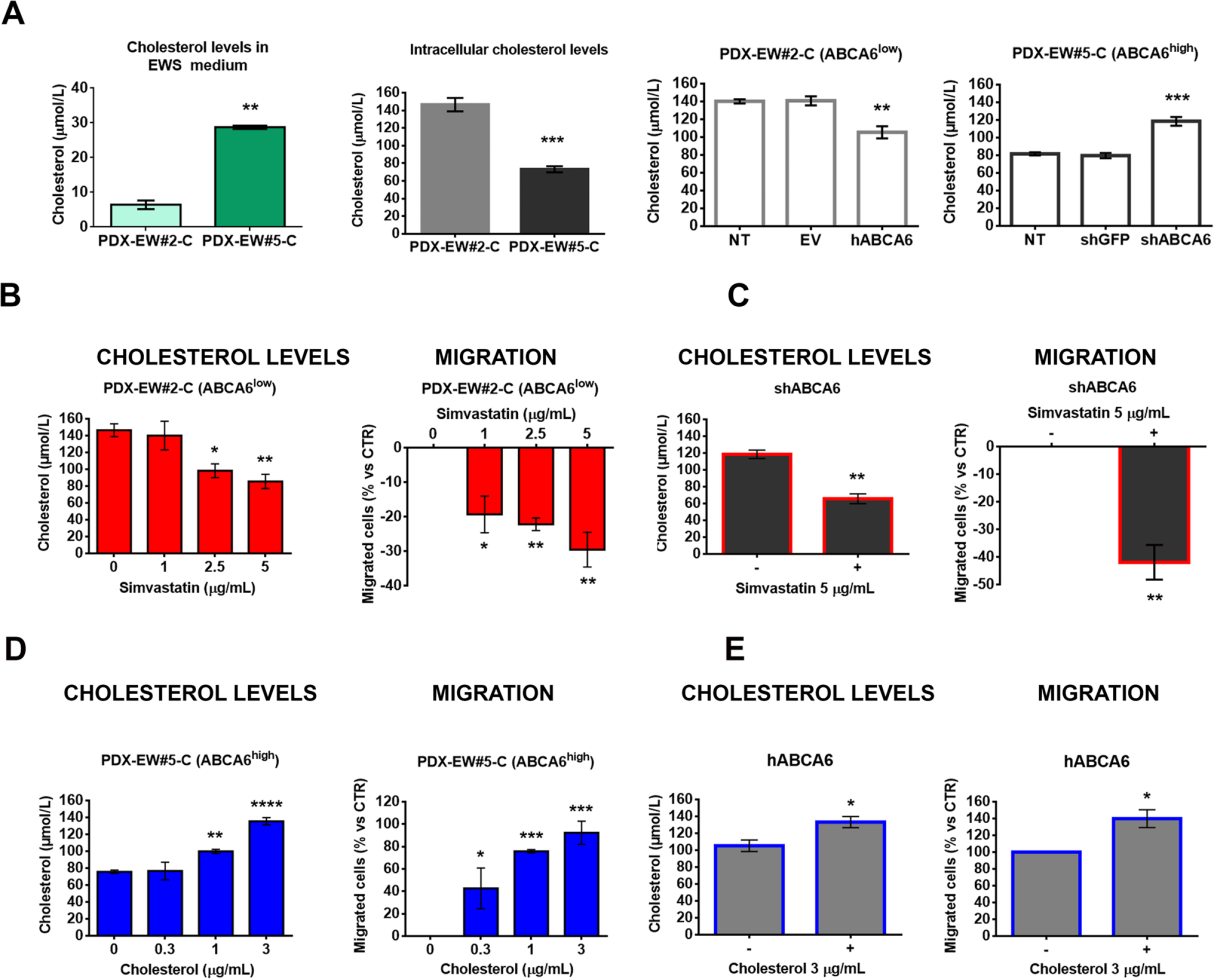
Impact of intracellular levels of cholesterol on EWS cell malignancy. **a**, Total cholesterol quantification by using a colorimetric assay in PDX-EW#2-C cells (ABCA6^low^) and PDX-EW#5-C cells (ABCA6^high^), both in the medium and at the intracellular level. Data are the mean ± SEM (*n* = 3); **p < 0.01; ***p < 0.001, Student’s t-test. Intracellular cholesterol was also quantified in PDX-EW#2-C cells after forced expression and in PDX-EW#5-C cells after silencing of ABCA6. Data are the mean ± SEM (*n* = 3); **p < 0.01; ***p < 0.001, one-way ANOVA. PDX-EW#2-C cells (non-transfected cells, **b**) and PDX-EW#5-C silenced cells (**c**) were exposed to simvastatin (72 h). Graphs represent the effects on intracellular cholesterol levels (mean ± SEM; *n* = 3) and cell migratory ability (mean ± SEM; *n* = 3). *p < 0.05; **p < 0.01 versus untreated cells (control, one-way ANOVA or Student’s t test). PDX-EW#5-C (non-transfected cells, **d**) and PDX-EW#2-C after forced expression (**e**) are exposed to exogenous cholesterol (72 h). Graphs represent the effects on intracellular cholesterol levels (mean ± SEM; *n* = at least 3) and cell migratory ability (mean ± SEM; *n* = 3). *p < 0.05; **p < 0.01; ***p < 0.001; ****p < 0.0001 versus untreated cells (control, one-way ANOVA or Student’s t test)

Members of the ABCA subfamily of transporters are thought to play important roles in lipid transport and trafficking, including the regulation of cholesterol efflux from cells (for a review, see [3]). Thus, both cholesterol intracellular levels and the amount released into the culture medium were measured in PDX-derived EWS cells with differential expression levels of ABCA6. We observed reduced intracellular levels of cholesterol and higher cholesterol contents in the medium of the less aggressive PDX-EWS#5-C cells (ABCA6^high^) compared to PDX-EWS#2-C cells (ABCA6^low^) (Fig. 4a). Consistently, decreased or enhanced cholesterol levels were measured in ABCA6-overexpressing or -silenced cells, respectively, compared to non-transfected controls (NT, Fig. 4a). Staining of cells with filipin III, a fluorescent dye that specifically stains membrane-bound cholesterol [25], confirmed the reduced intracellular levels of cholesterol in the less aggressive PDX-EWS#5-C cells (ABCA6^high^; Supplementary Fig. 3). In contrast, in the PDX-EWS#2-C cells (ABCA6^low^), cholesterol was well detectable at the cell surface membrane and at the intracellular level (Supplementary Fig. 3). Functional relationships between ABCA6 expression, intracellular levels of cholesterol and malignant features of EWS cells were confirmed by exposing cells to simvastatin, a well-known inhibitor of the rate-limiting enzyme 3-hydroxy-3-methylglutaryl-CoA reductase (HMG-CoA reductase) in the cholesterol synthesis pathway [26], or exogenous cholesterol. In cells characterized by low expression of ABCA6 and high intracellular levels of cholesterol (PDX-EWS#2-C or PDX-EWS#5-C cells silenced), treatment with simvastatin diminished the cellular content of cholesterol, as expected, and weakened the migration abilities of cells in a dose-dependent manner (Fig. 4b and c). In contrast, the exposure of cells characterized by high expression of ABCA6 and low intracellular levels of cholesterol (PDX-EWS#5-C cells or PDX-EWS#2-C cells forced for ABCA6 expression) to exogenous cholesterol increased the intracellular levels of the lipid and induced a dose-dependent enhancement of migrated cells (Fig. 4d and e). As further confirmation, the inhibitory effect of simvastatin on ABCA6^low^ cell migration was completely reversed by exogenous cholesterol (Supplementary Fig. 4).

**Fig. 5:**
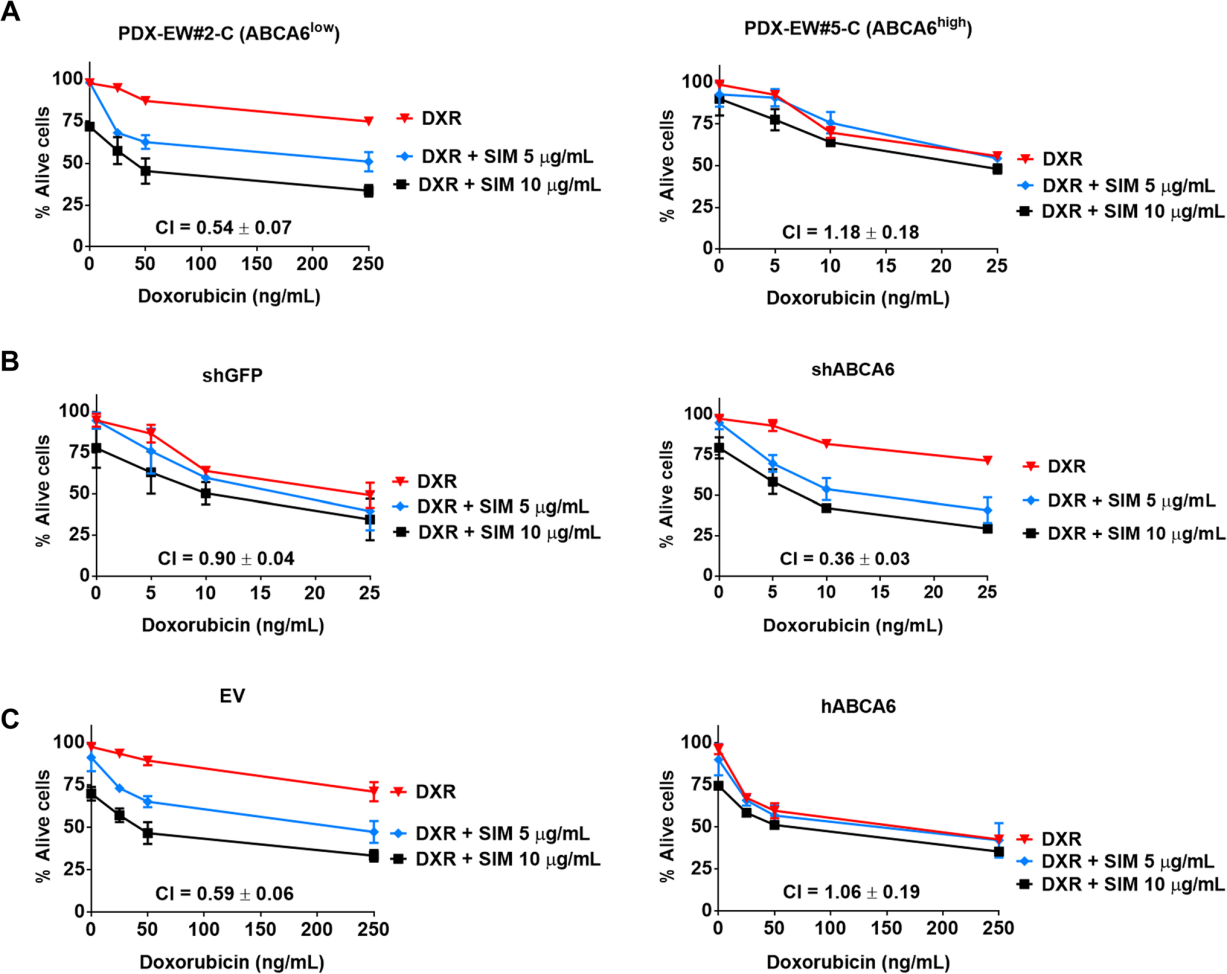
Combined effects of simvastatin and doxorubicin on EWS cell growth. Cells were exposed to different doses of simvastatin (SIM) for 72 h before being exposed to doxorubicin (DXR) alone or in combination for an additional 24 h in: **a**, PDX-EW#2-C (ABCA6^low^) and PDX-EW#5-C (ABCA6^high^); **b**, PDX-EW#5-C after ABCA6 silencing; **c**, PDX-EW#2-C after forced expression of ABCA6. Cell growth was evaluated by vital cell count. CI: combination index (synergism: CI < 0.90; additive 0.9 ≤ CI ≤ 1.1; antagonism: CI > 1.1)

Pretreatment with simvastatin also increased the sensitivity to doxorubicin in the most aggressive ABCA6^low^ cells. Indeed, a clear synergistic effect between the two drugs was observed in ABCA6^low^ cells, but not in the less aggressive ABCA6^high^ cells (Fig. 5a). Additionally, while the silencing of ABCA6 expression in PDX-EW#5-C (ABCA6^high^) cells induced synergism (Fig. 5b right), the forced expression of ABCA6 in PDX-EW#2-C (ABCA6^low^) cells partly reversed the favoring effect of simvastatin (Fig. 5c right). This implies that cholesterol lowering drugs should be especially considered for the treatment of the most aggressive cells characterized by low expression of the transporter. Accordingly, the PDX-EW#2-C (ABCA6^low^) cells were found to be more sensitive to simvastatin than the PDX-EW#5-C (ABCA6^high^) cells (IC50 values = 63.65 ± 5.47 µg/ml vs 188.99 ± 16.82 µg/ml, respectively; *p* = 0.0021, Student’s t-test).

### ABCA6-mediated variations in intracellular levels of cholesterol impair activation of IGF1R/AKT/mTOR signaling while promoting doxorubicin-induced apoptosis

**Fig. 6:**
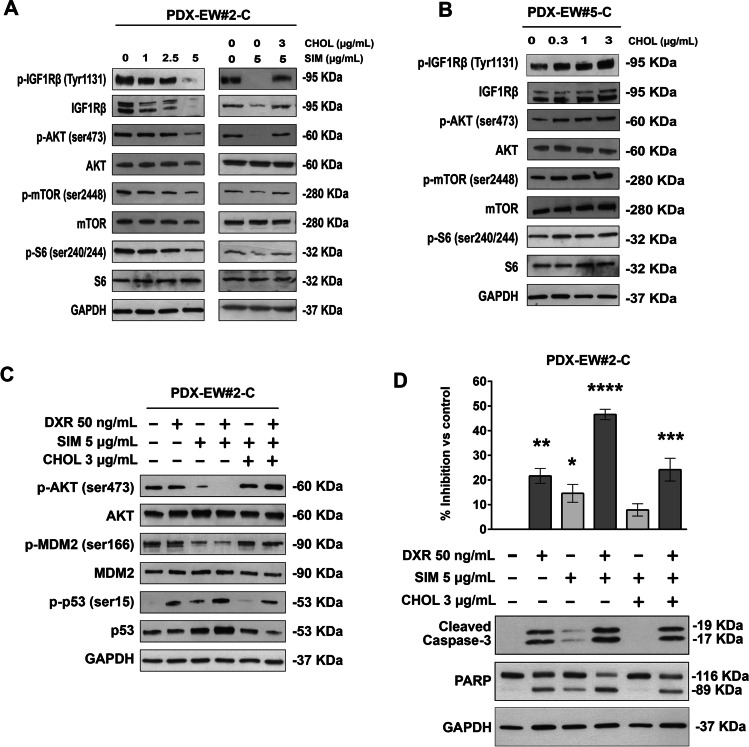
Cholesterol mediates activation of IGF1R/AKT signaling and doxorubicin-induced apoptosis. **a,** IGF1R pathway inhibition in PDX-EW#2-C (ABCA6^low^) cells treated with increasing doses of simvastatin (SIM) and after the rescue effect of exogenous cholesterol (CHOL) exposure. A representative western blot of three is shown. GAPDH was used as a loading control. **b**, IGF1R pathway induction in PDX-EW#5-C (ABCA6^high^) cells after exogenous cholesterol exposure. A representative western blot of three is shown. GAPDH was used as a loading control. **c**, AKT/MDM2/p53 proapoptotic pathway activation in PDX-EW#2-C cells after doxorubicin (DXR, 3 h) exposure alone or in combination with SIM or SIM plus CHOL. A representative western blot of three is shown. GAPDH was used as a loading control. **d**, Growth inhibition and apoptosis induction represented by caspase 3 and PARP cleavage, after doxorubicin (DXR, 24 h) exposure alone or in combination with SIM or SIM plus CHOL. Data in the graph are the mean ± SEM (*n* = 3); *p < 0.05; **p < 0.01; ***p < 0.001, ****p < 0.0001. One–way ANOVA versus control (nontreated cells). A representative western blot of three is shown. GAPDH was used as a loading control

To illustrate the mechanisms that connect the expression of ABCA6 and the levels of cholesterol to cell migration and drug resistance, we treated the most aggressive PDX-EWS#2-C cells (ABCA6^low^) with simvastatin, while the less aggressive PDX-EWS#5-C (ABCA6^high^) cells were exposed to exogenous cholesterol. Previous studies have shown that cholesterol-lowering drugs could inhibit AKT signaling in cancer cells by downregulating IGF1R expression [27, 28]. In our experimental model, we found that (i) transient overexpression or silencing of ABCA6 modulated AKT phosphorylation (Supplementary Fig. 5) and (ii) incubation of PDX-EWS#2-C cells with simvastatin (1–5 μg/ml) decreased the constitutive level of AKT phosphorylation on residue Ser473 (Fig. 6a). The effect lasted at least 72 h and was associated with upstream lower expression and phosphorylation on residue Tyr1131 of IGF1R, a key participant in EWS development and progression [29] and reduced downstream phosphorylation of mTOR on residue Ser2448 and ribosomal protein S6 on residue Ser240/244 (Fig. 6a). The simvastatin-induced IGF1R/AKT/mTOR inhibition in PDX-EWS#2-C cells was reversed by exogenous cholesterol (Fig. 6a). Accordingly, exposure of PDX-EWS#5-C (ABCA6^high^) cells to cholesterol stimulated phosphorylation of IGF1R and downstream mediators (Fig. 6b). Previous studies [30–32] have shown that cholesterol modification disrupts lipid raft domains, including caveolae [33] and alters the interaction among lipid raft-associated proteins. We here confirmed that overexpression of ABCA6 in PDX-EWS#2-C cells or their exposure to simvastatin led to decreased expression of caveolin-1, while ABCA6 silencing in PDX-EWS#5-C cells or their exposure to exogenous cholesterol led to increased levels of caveolin-1 on the cell surface (Supplementary Fig. 6). Although it is beyond the purpose of this study, a deeper evaluation of the cholesterol-caveolin-IGF1R signaling interactions, IGF1R being a resident of lipid rafts and caveolae [30, 31], it is very likely that cholesterol depletion followed by ABCA6 high expression could inhibit IGF1R and AKT signaling. This implies that the higher content of cholesterol, which characterizes EWS cells with low expression of the ABCA6 transporter, favors the constitutive IGF1R/AKT/mTOR signaling activation that is known to be sustained by the autocrine production of IGF1 [34, 35]. Accordingly, a comparison between untreated PDX-EWS#2-C and PDX-EWS#5-C cells clearly showed the highest constitutive signaling activation in the cell line with a lower expression of ABCA6, a higher content of cholesterol and a more aggressive phenotype (Fig. 6a and b). In PDX-EW#2-C cells, simvastatin, besides hampering AKT signaling also reduced Ser 166 phosphorylation of MDM2 (Fig. 6c), leading to increased p53 activation [36]. The effects on MDM2/p53 were further increased when simvastatin-pretreated cells were exposed to doxorubicin (Fig. 6c). The pro-apoptotic influence of p53 on the statin-induced sensitizing effect was confirmed by caspase 3 and PARP cleavage (Fig. 6d). Exposure to exogenous cholesterol antagonized these effects on proapoptotic signaling induced by the combination of doxorubicin with simvastatin (Fig. 6c, d). The ability of simvastatin to sensitize cells to doxorubicin-induced apoptosis mirrored the inhibitory drug effects on cell growth (Fig. 6d), further supporting the therapeutic utility of lowering cholesterol levels in the most aggressive EWS cells.

## Discussion

In this study, we provide evidence that localized EWS patients who underwent conventional multidrug chemotherapy have a more favorable course of disease when tumoral expression of the *ABCA6* transporter is high. These clinical data obtained in three independent cohorts of EWS patients are in conformity with a recent report on pancreatic ductal adenocarcinoma [37] and a previous observation in neuroblastoma [38], but in sharp contrast with the general idea that overexpression of ABC transporters is associated with more pronounced malignancy and drug resistance due to their capabilities to export drugs or toxins (through their canonical function of detoxification) [39]. Here, we show that expression of ABCA6 impairs the intracellular levels of cholesterol, which is an important component of cellular membranes reported to regulate membrane fluidity and functionality [40]. Cancer cells accumulate cholesterol, particularly in the cytosolic face of the plasma membrane, where cholesterol offers structural support and strongly affects the stability and function of growth factor receptors, integrins and cell surface glycoproteins. Consequently, the intracellular cholesterol content is involved in the control over major biological processes such as endocytosis, intracellular signaling pathway activation, cell adhesion and motility (for a review, see [41]). Depletion of cholesterol in cancer cells has been reported to reduce tumor cell migration [42] and to increase sensitivity to chemotherapeutic agents [43]. Here, we report that when the ABCA6 transporter is highly expressed, either constitutively or after being forced, cells exhibit low intracellular levels of cholesterol and a reduced capability to migrate, and they are more sensitive to doxorubicin and other DNA-damaging agents, such as etoposide and ifosfamide. In contrast, EWS cells that constitutively express low levels of ABCA6 or that have been silenced for the expression of this transporter have higher levels of intracellular cholesterol and display a more malignant phenotype. Furthermore, higher levels of cholesterol were detected in the culture medium of less aggressive cells (ABCA6^high^) indicating increased efflux of cholesterol. Exposure to statins, which suppress intracellular cholesterol synthesis through the inhibition of HMG-CoA reductase [26], or to exogenous cholesterol modulate cell behavior accordingly. In the exploration of the underlying mechanisms, we found that cells characterized by high expression of the transporter and lower levels of cholesterol showed decreased constitutive activation of IGF1R/AKT signaling compared to ABCA6 low expressors that present a higher lipid content. Accordingly, the cholesterol lowering drug simvastatin was found to inhibit IGF1R/AKT/mTOR activation, whereas exogenous cholesterol stimulated upregulation and activation of IGF1R and phosphorylation of AKT/mTOR. This finding is in line with previous studies showing that the activation of IGF1R depends critically on the levels of cholesterol [30–32], which may alter interactions between lipid rafts, including caveolae, and associated tyrosine kinase receptors (for a review see [44]). In particular, it was found that IGF1R phosphorylation is inhibited by cholesterol depletion, while it is restored by the replacement with exogenous cholesterol [32]. Other studies reported an oncogenic and pro-metastatic role of caveolin-1 in EWS [45–47] and how IGF1R signaling is enhanced after caveolin-mediated receptor internalization [48, 49]. Our data, reporting the role of ABCA6 in diminishing intracellular cholesterol, decreasing caveolin-1 expression and inhibiting IGF1R signaling, are consistent with this evidence and support the hypothesis that the ability to form ordered domains is sufficient to support activation of IGF1R signaling and tumor growth. In addition, we provide evidence that through inhibition of IGF1R/AKT signaling, statins also prevent Ser166 phosphorylation of MDM2, leading to an increased p53 response and enhanced pro-apoptotic effects of doxorubicin. Of note, highly malignant EWS cells that express low levels of ABCA6 and have higher levels of intracellular cholesterol are more sensitive to statins. Additionally, pretreatment with simvastatin synergistically increased cell sensitivity to doxorubicin. This finding supports the possible use of statins as adjuvant agents in therapy against EWS. Statins are commonly administered to treat atherosclerotic cardiovascular disease, but they also exert pro-apoptotic, anti-angiogenic and immunomodulatory effects in various tumor cell types (for a review, see [50, 51]) and decrease the development of multidrug resistance in vitro (for a review, see [52]). At the clinical level, however, the results related to the use of statins or other cholesterol-lowering drugs are controversial, with some studies suggesting prolonged survival and others reporting no benefit (for a review, see [53]). In this study, we highlight how the expression of ABCA6 may affect the sensitivity to statins, thereby supporting the idea that evaluation of the expression level of these transporters is necessary to identify patients who may benefit from the anticancer effects of statins.

## Conclusion

**Fig. 7:**
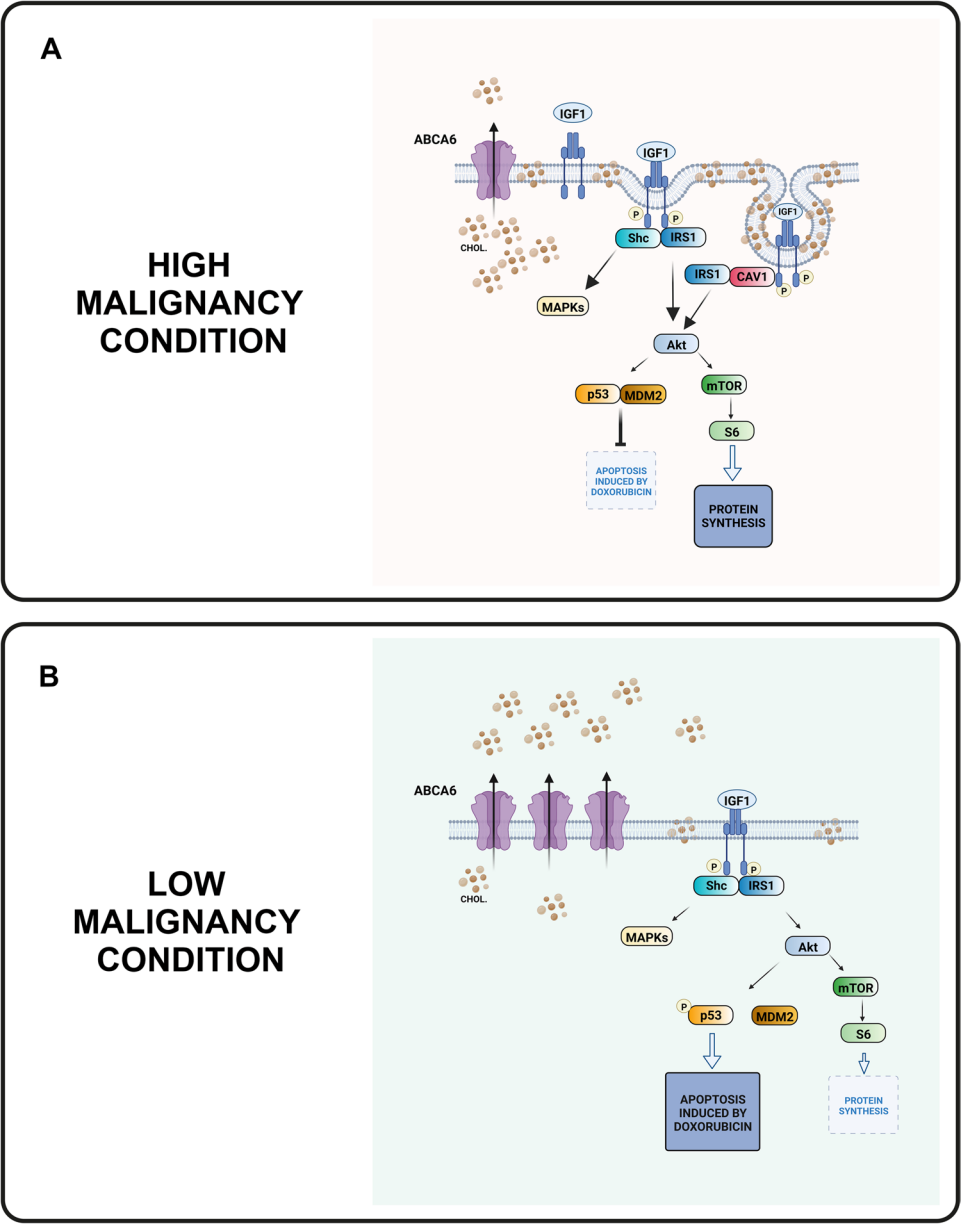
Simplified representation of the relationships between ABCA6, cholesterol (CHOL), and IGF1R signaling in EWS cells in the high malignancy condition (ABCA6^low^) or the lower aggressive status (ABCA6^high^). The proposed mechanism is summarized as follows: **a**, when cells express low ABCA6, cellular levels of cholesterol are increased with consequent enrichment in caveolae and IGF1R signaling. Co-localization of IGF1R and caveolin-1 together with enhanced caveolin-dependent IGF1R signaling are well-demonstrated [31, 48, 49]. The sustained activation of IGF1R/AKT triggers both mTOR/S6 signaling, which leads to increased protein synthesis and cell survival, and MDM2. MDM2 phosphorylated at Ser166 favors p53 degradation, which results in an attenuated p53-mediated apoptotic response to DNA-damaging agents. **b**, when cells express high levels of ABCA6, cholesterol efflux is increased, thereby diminishing the total content of cholesterol in the cells and reducing IGF1R/AKT signaling. This prevents the phosphorylation of MDM2 at Ser166, leading to an increased p53 response, while downstream inhibition of mTOR/S6 decreases cell survival. (The images were “Created with BioRender.com)

Our data indicate that most aggressive EWS are characterized by a lower expression of ABCA6, a condition that facilitates the progression of EWS by keeping cellular cholesterol at high levels. This results in an enrichment of caveolae, sustained activation of IGF1R/AKT signaling, increased function of MDM2 and attenuated p53 responses to DNA-damaging agents. In contrast, high ABCA6 expression was found to be associated with a better patient’s prognosis and, at the cellular level, with improved chemotherapy-induced apoptosis likely due to decreased levels of cholesterol and reduction of pro-survival signaling (Fig. 7). This finding indicates a selective vulnerability of EWS cells and sustains ABCA6 evaluation for a rationale use of statins as adjuvant drugs. Overall, our study highlights the importance of ABCA6 in cancer and provides a mechanistic explanation for its involvement in the regulation of cancer aggressiveness.

## Supplementary Information

Below is the link to the electronic supplementary material.

ESM 1 (PNG 290 kb)

High Resolution (TIF 2746 kb)

ESM 2 (PNG 91 kb)

High Resolution (TIF 673 kb)

ESM 3 (PNG 345 kb)

High Resolution (TIF 2145 kb)

ESM 4 (JPG 607 kb)

High Resolution (TIF 5633 kb)

ESM 5 (PNG 282 kb)

High Resolution (TIF 3542 kb)

ESM 6 (PNG 1934 kb)

High Resolution (TIF 32278 kb)

ESM 7 (DOCX 57.9 kb)

ESM 8 (DOCX 31 kb)

ESM 9 (DOCX 25 kb)

ESM 10 (DOCX 35 kb)

ESM 11 (DOCX 34 kb)

ESM 12 (DOC 33 kb)

ESM 13 (DOCX 29 kb)

## Data Availability

All data generated or analyzed during this study are included in this manuscript and its supplementary files.
