# Correction to: ABCA6 affects the malignancy of Ewing sarcoma cells via cholesterol-guided inhibition of the IGF1R/AKT/MDM2 axis

**DOI:** 10.1007/s13402-022-00735-z

**Published:** 2022-11-09

**Authors:** Michela Pasello, Anna Maria Giudice, Camilla Cristalli, Maria Cristina Manara, Caterina Mancarella, Alessandro Parra, Massimo Serra, Giovanna Magagnoli, Florencia Cidre-Aranaz, Thomas G. P. Grünewald, Carla Bini, Pier-Luigi Lollini, Alessandra Longhi, Davide Maria Donati, Katia Scotlandi

**Affiliations:** 1 Experimental Oncology Laboratory, IRCCS Istituto Ortopedico Rizzoli, 40136 Bologna, Italy; 2 Alma Mater Institute On Healthy Planet - Alma Healthy Planet, University of Bologna, Bologna, Italy; 3 Department of Experimental, Diagnostic and Specialty Medicine (DIMES), University of Bologna, Bologna, Italy; 4 Department of Pathology, IRCCS Istituto Ortopedico Rizzoli, Bologna, Italy; 5 Division of Translational Pediatric Sarcoma Research, German Cancer Research Center (DKFZ), German Cancer Consortium (DKTK), Heidelberg, Germany; 6 Hopp-Children’s Cancer Center (KiTZ), Heidelberg, Germany; 7 Institute of Pathology, Heidelberg University Hospital, Heidelberg, Germany; 8 Laboratory of Forensic Genetics, Department of Medical and Surgical Sciences, University of Bologna, Bologna, Italy; 9 Osteoncologia, Sarcomi dell’osso e dei Tessuti Molli e Terapie Innovative, IRCCS Istituto Ortopedico Rizzoli, Bologna, Italy; 10 Unit of 3rd Orthopaedic and Traumatologic Clinic Prevalently Oncologic, IRCCS Istituto Ortopedico Rizzoli, Bologna, Italy; 11 Department of Biomedical and Neuromotor Sciences (DIBINEM), University of Bologna, Bologna, Italy

**Correction to: Cellular Oncology**

**https://doi.org/10.1007/s13402-022-00713-5**

In this article an incorrect affiliation had inadvertently been added. The original article has been updated.

