# Supplementary figures and images for: ABCA6 affects the malignancy of Ewing sarcoma cells via cholesterol-guided inhibition of the IGF1R/AKT/MDM2 axis
Source: Cell Oncol (Dordr). 2022 Sep 23;45(6):1237–51. doi: 10.1007/s13402-022-00713-5 (PMC9747862; doi:10.1007/s13402-022-00713-5)

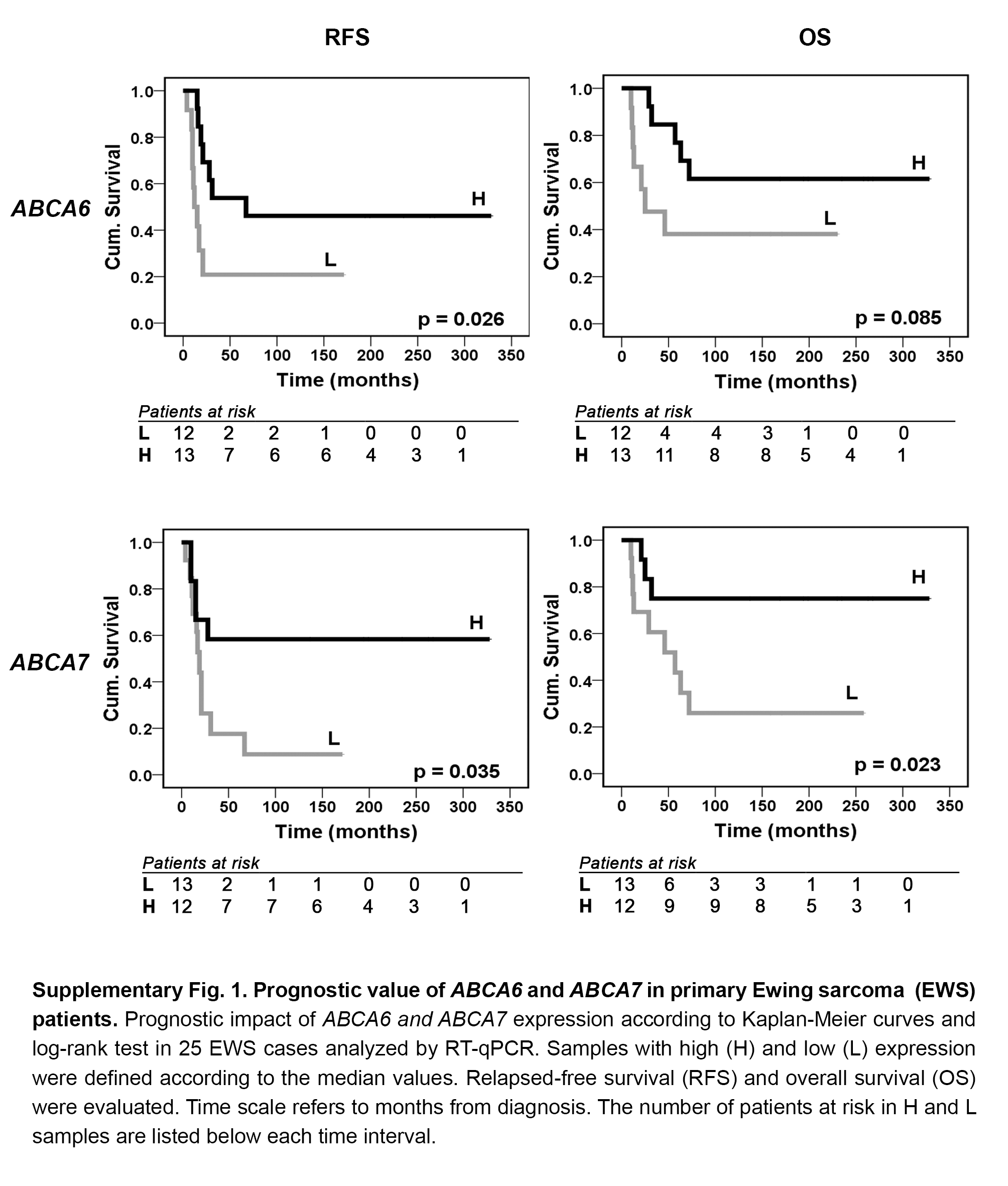

Supplement: Supplementary file 1 — (PNG 290 kb) [file 13402_2022_713_Fig8_ESM.png]

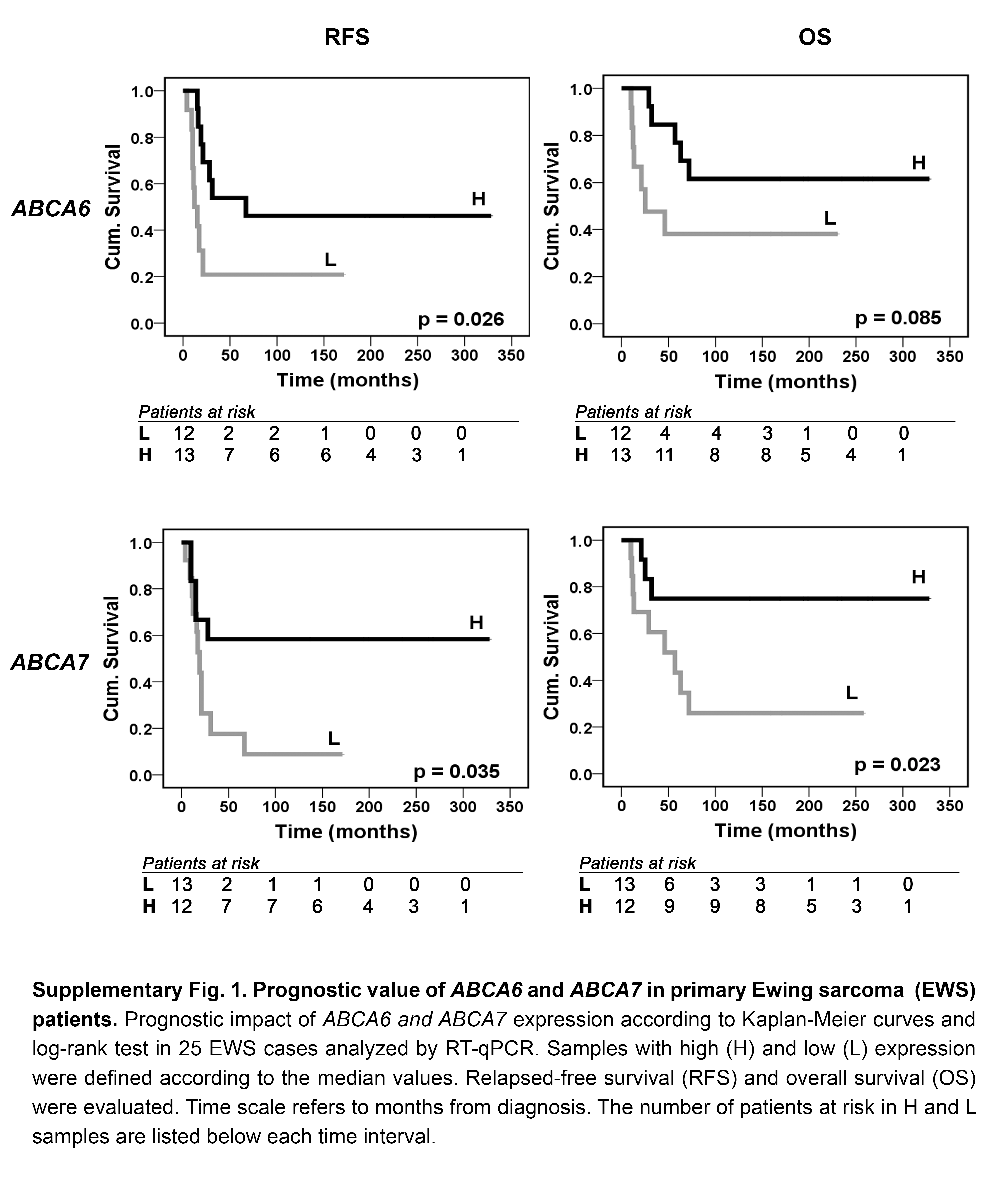

Supplement: Supplementary file 2 — High Resolution (TIF 2746 kb) [file 13402_2022_713_MOESM1_ESM.tif]

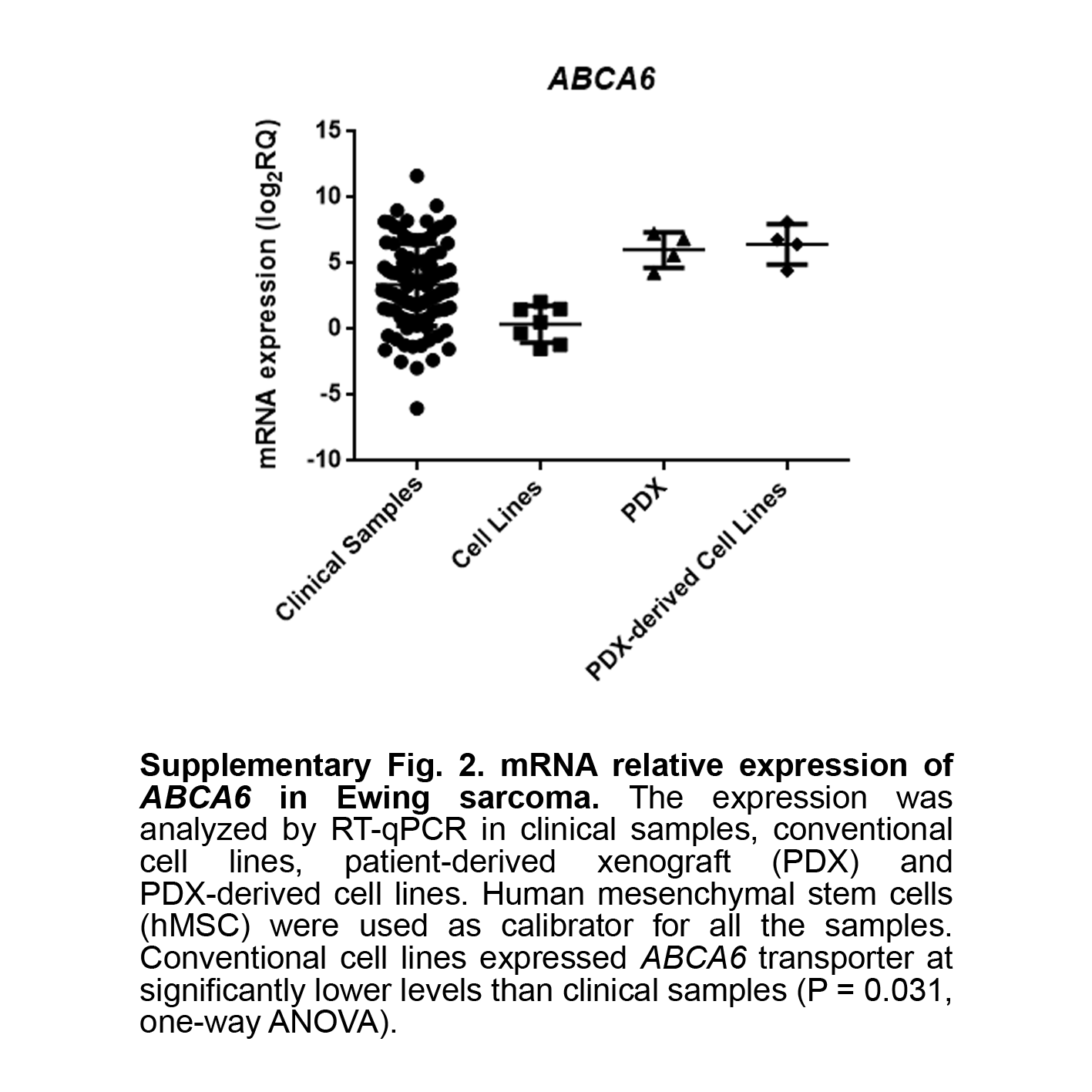

Supplement: Supplementary file 3 — (PNG 91 kb) [file 13402_2022_713_Fig9_ESM.png]

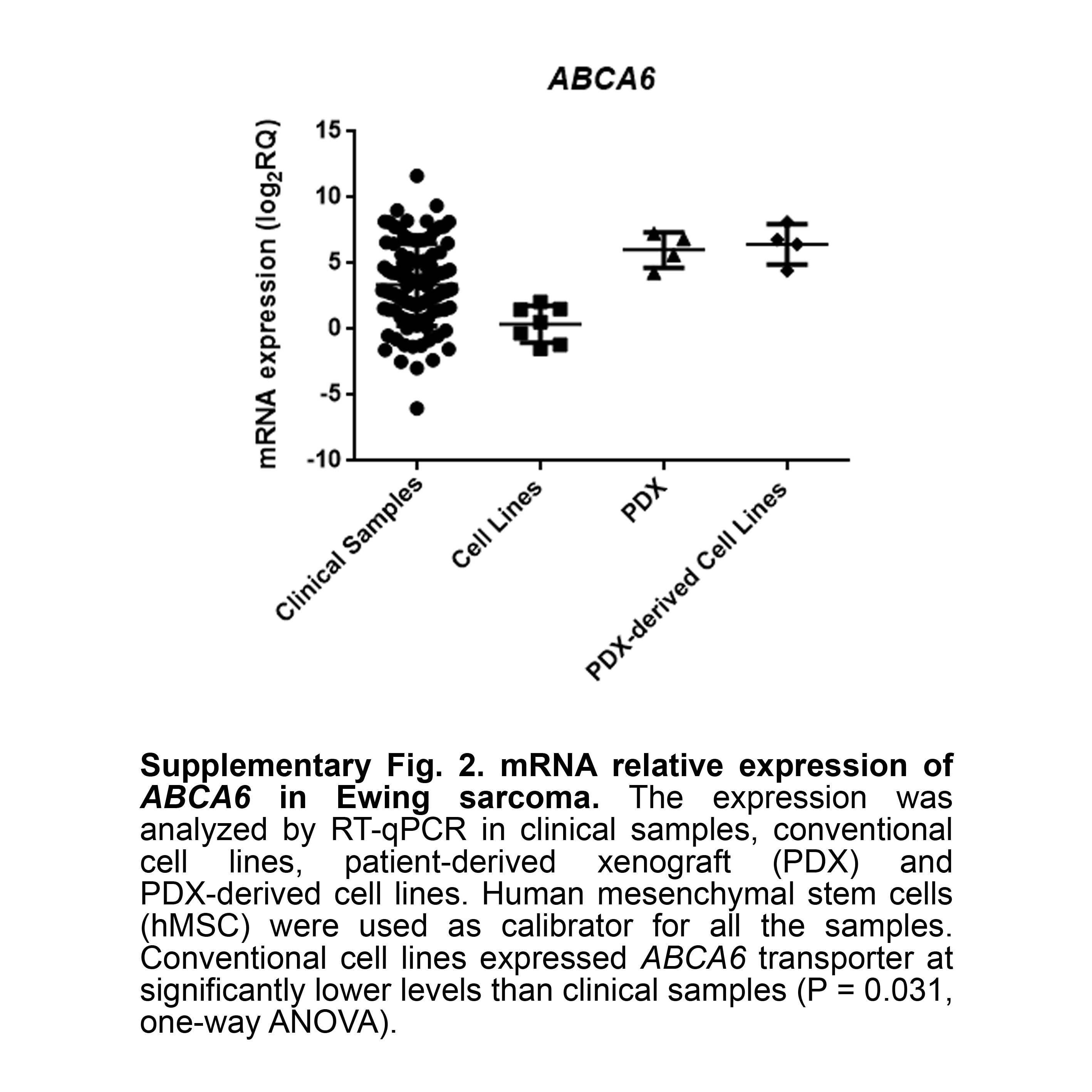

Supplement: Supplementary file 4 — High Resolution (TIF 673 kb) [file 13402_2022_713_MOESM2_ESM.tif]

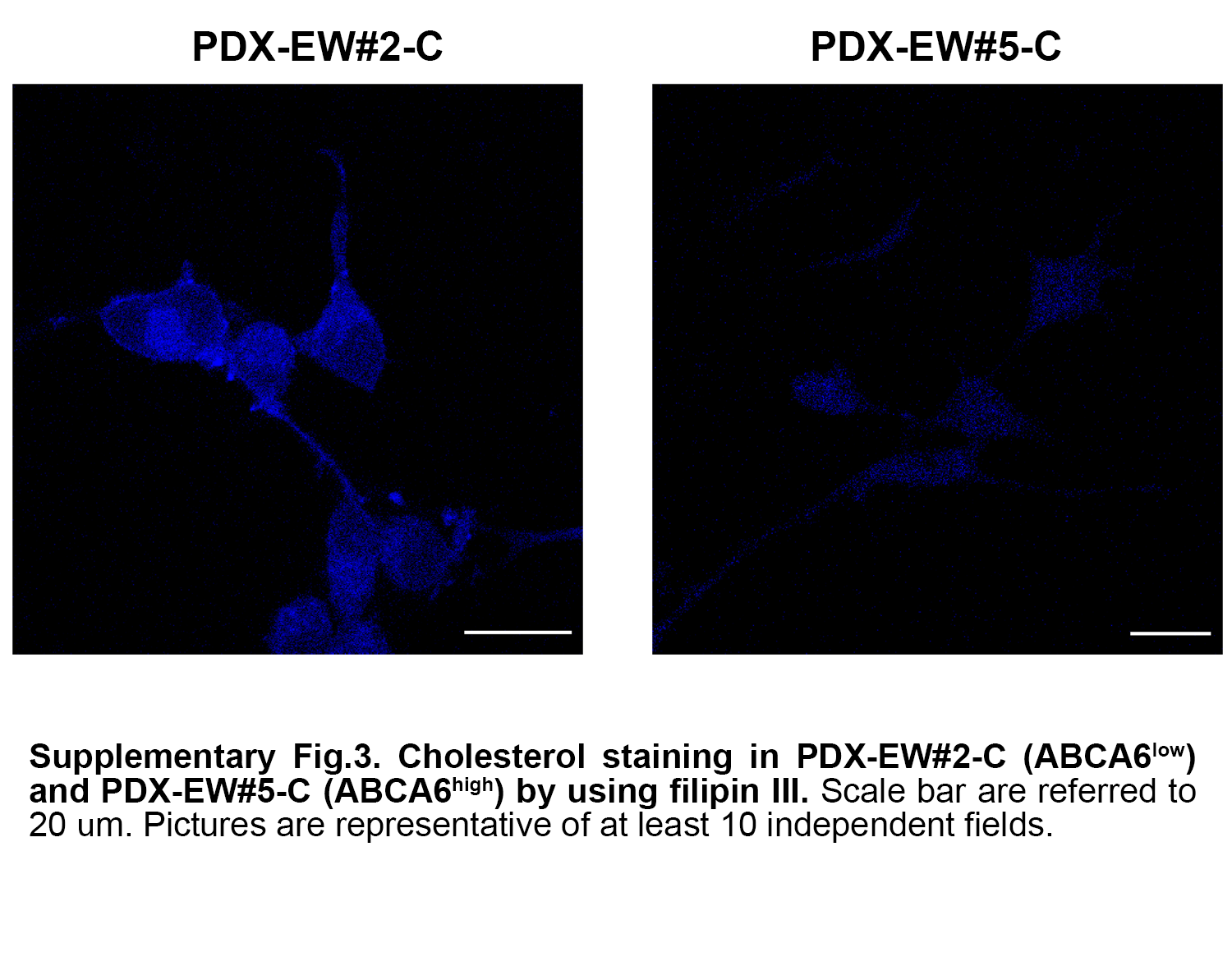

Supplement: Supplementary file 5 — (PNG 345 kb) [file 13402_2022_713_Fig10_ESM.png]

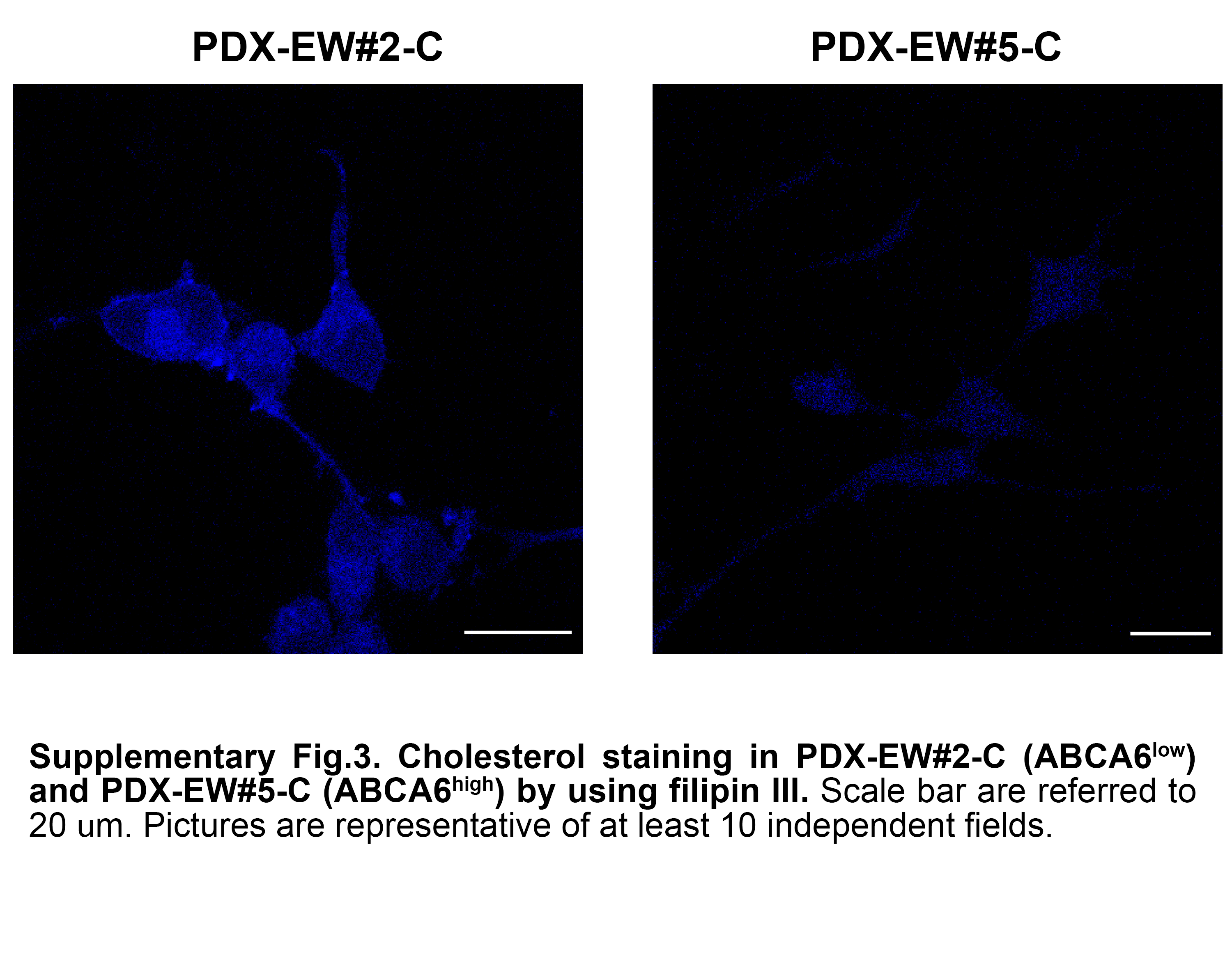

Supplement: Supplementary file 6 — High Resolution (TIF 2145 kb) [file 13402_2022_713_MOESM3_ESM.tif]

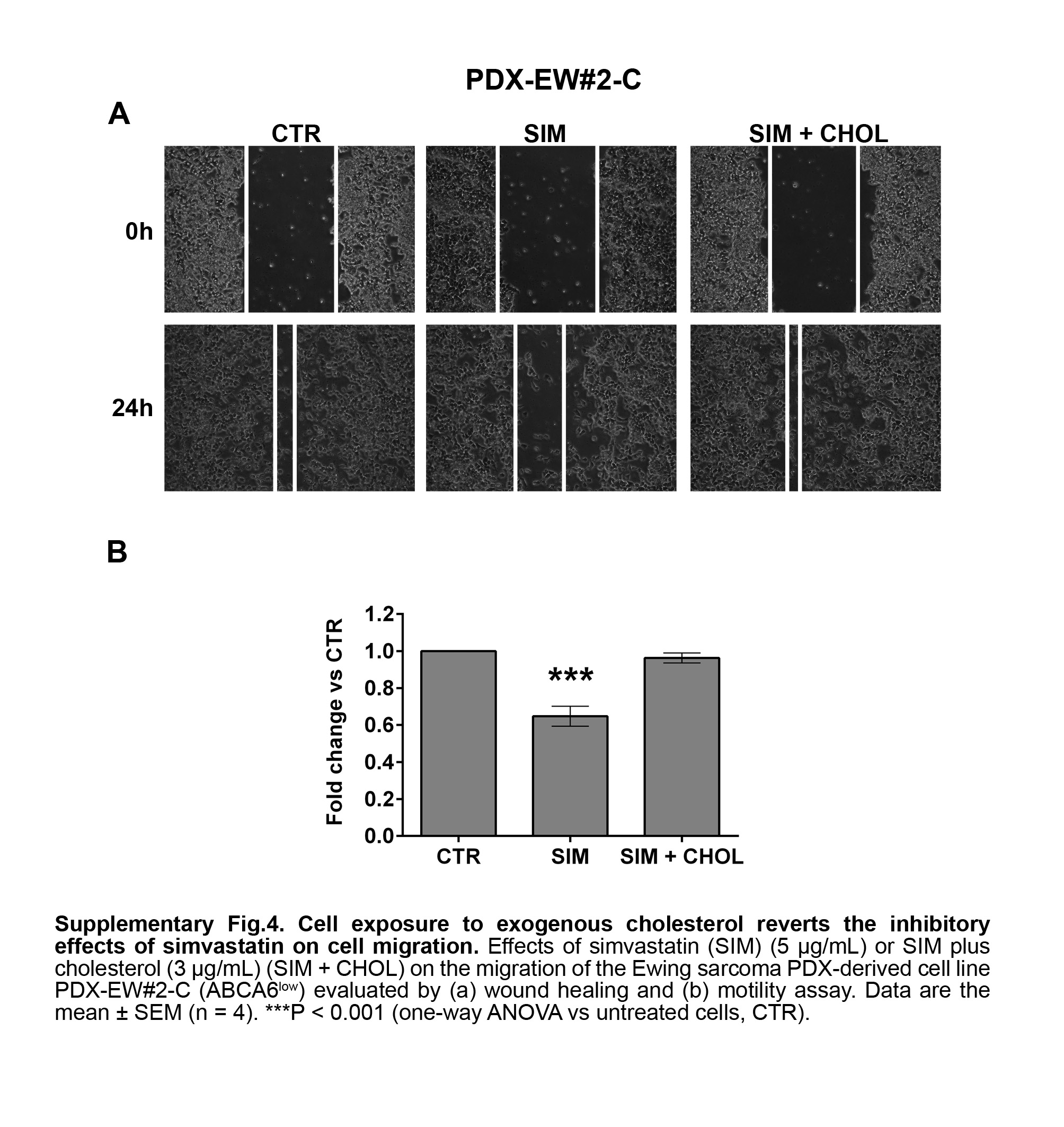

Supplement: Supplementary file 7 — (JPG 607 kb) [file 13402_2022_713_Fig11_ESM.jpg]

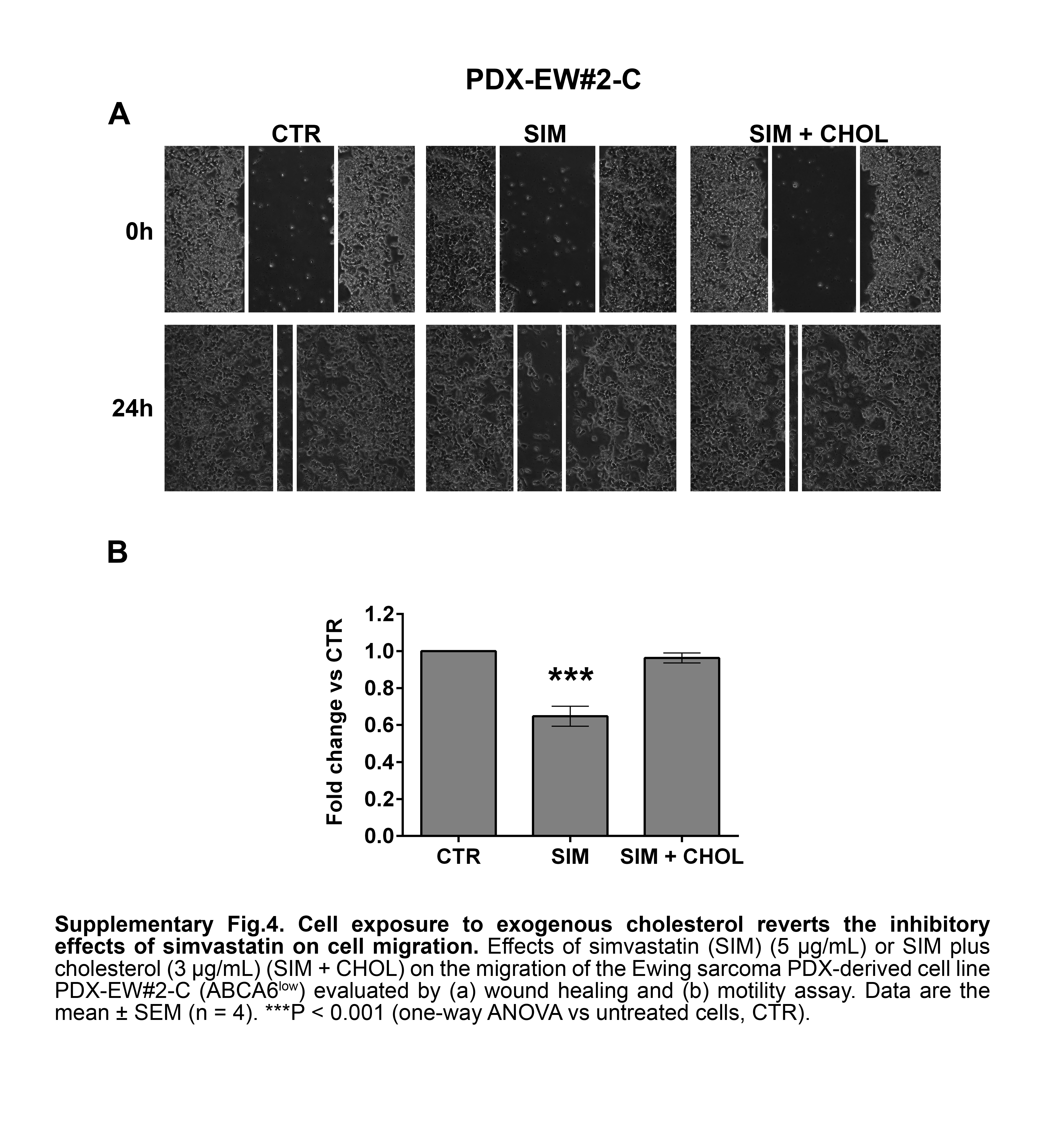

Supplement: Supplementary file 8 — High Resolution (TIF 5633 kb) [file 13402_2022_713_MOESM4_ESM.tif]

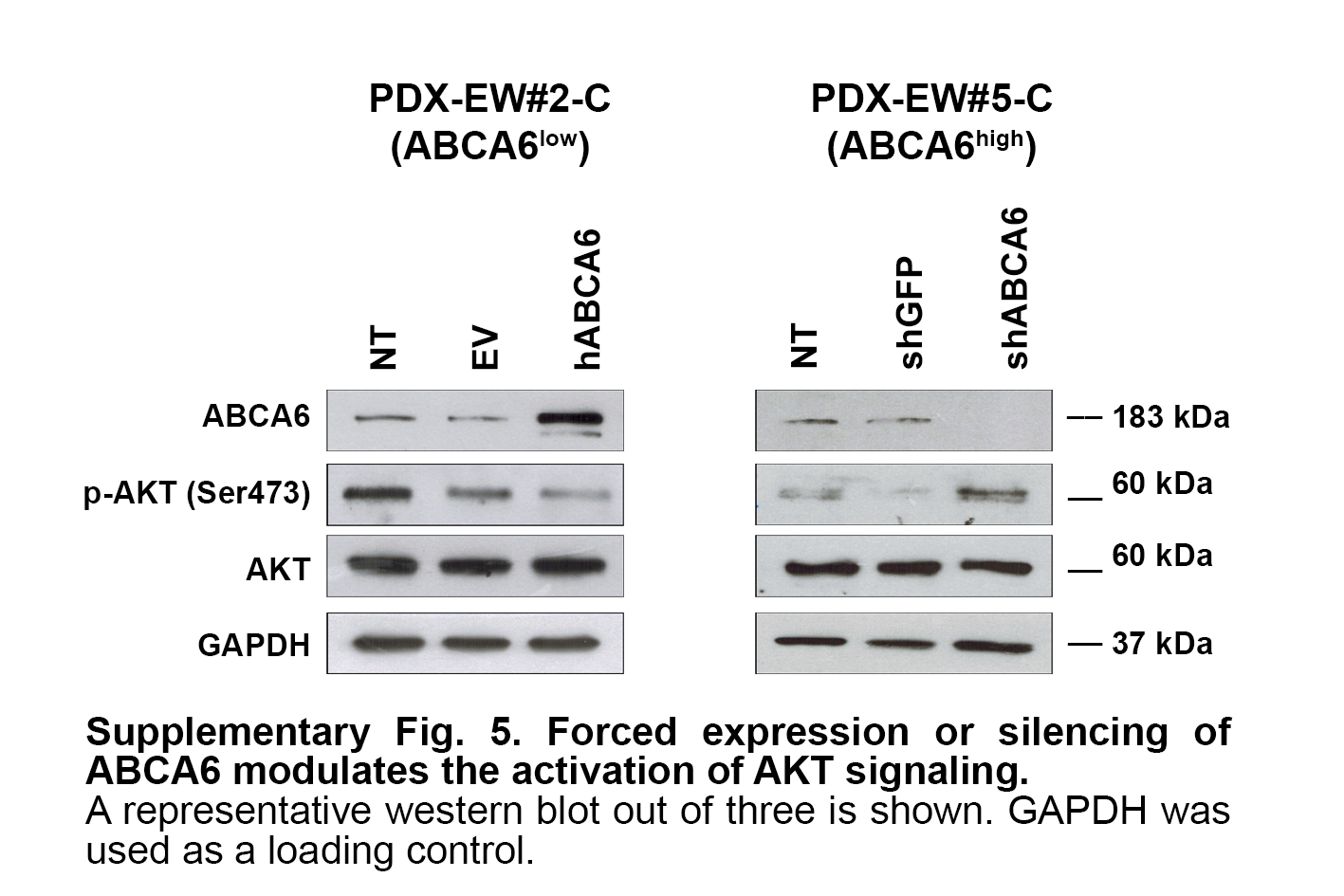

Supplement: Supplementary file 9 — (PNG 282 kb) [file 13402_2022_713_Fig12_ESM.png]

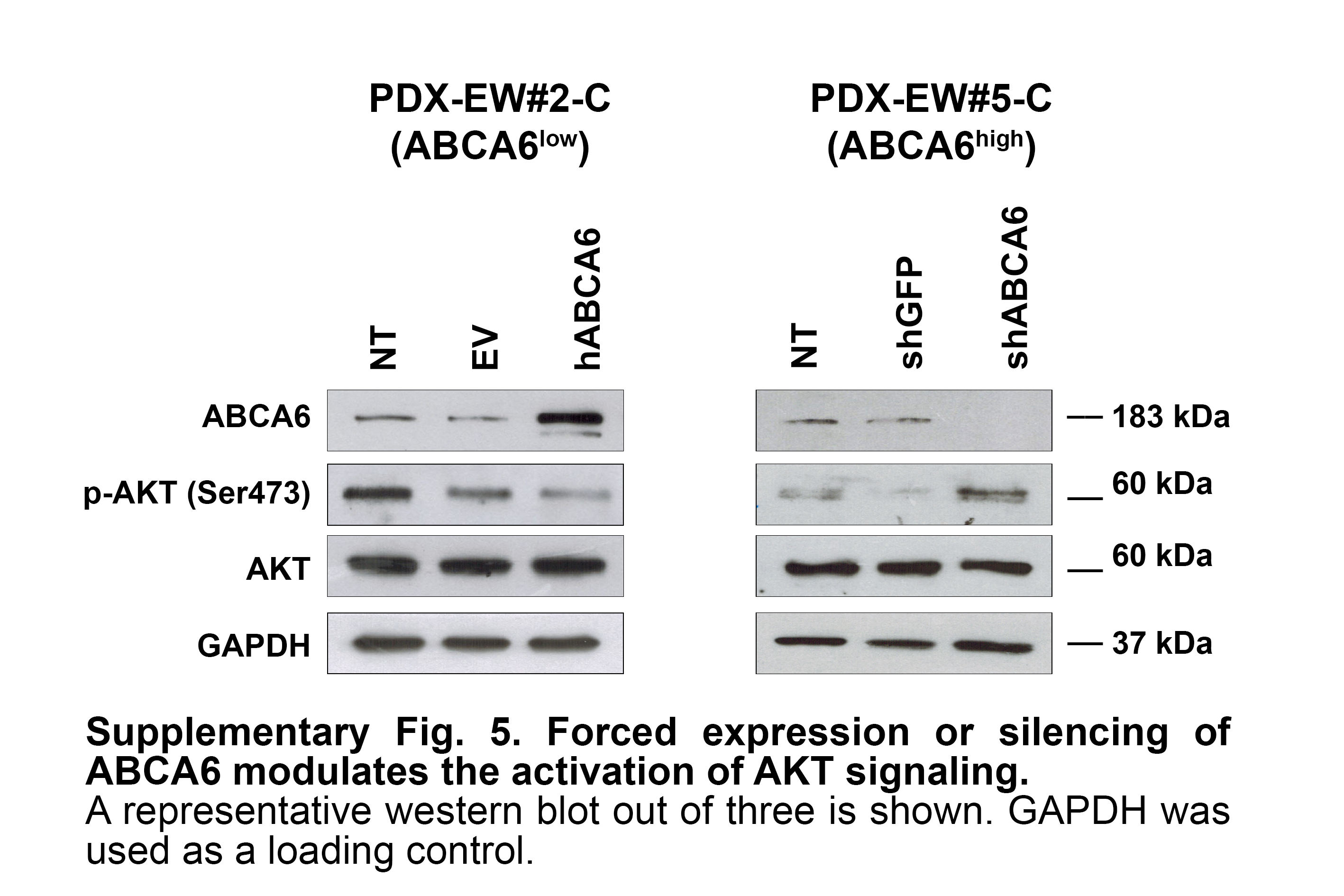

Supplement: Supplementary file 10 — High Resolution (TIF 3542 kb) [file 13402_2022_713_MOESM5_ESM.tif]

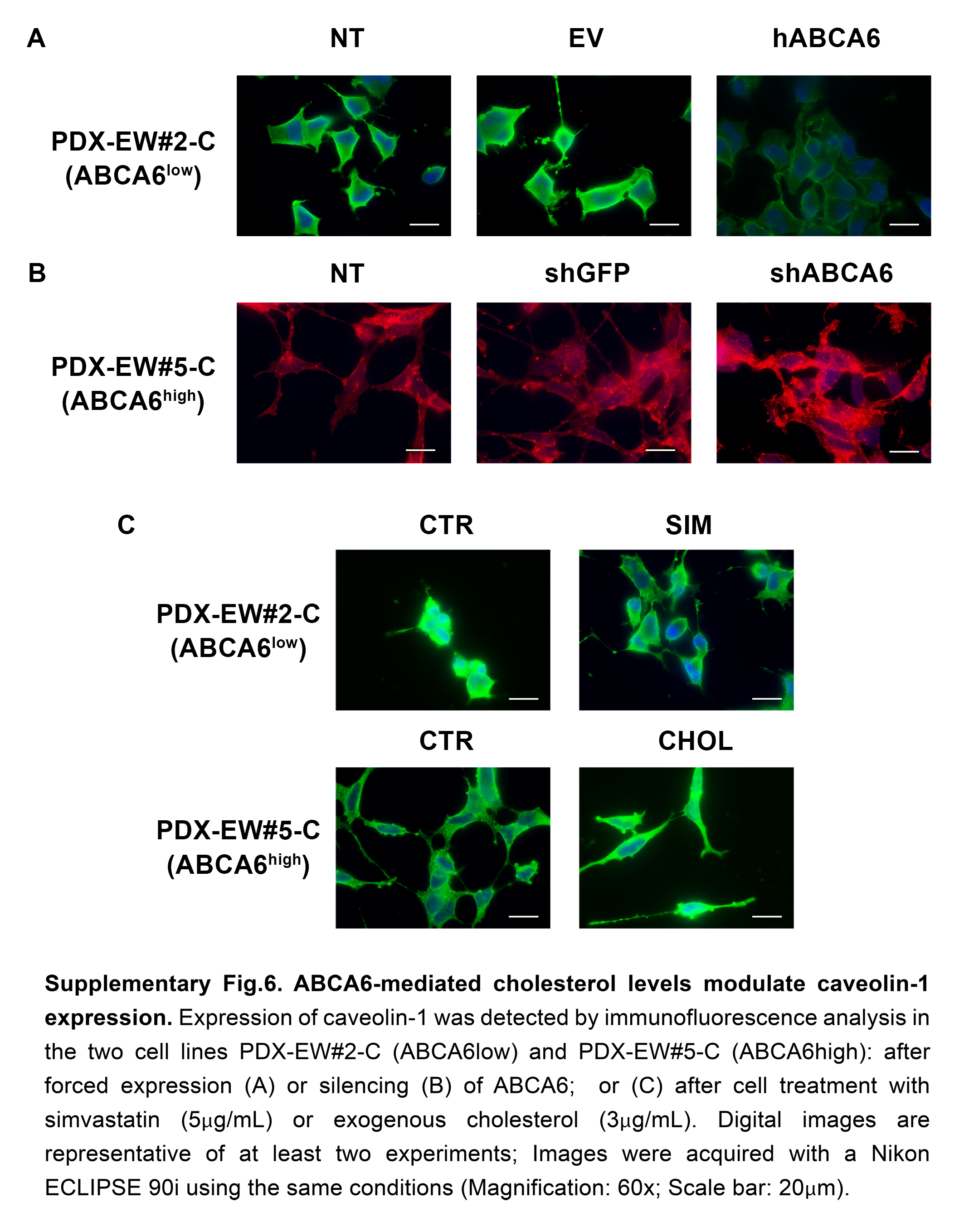

Supplement: Supplementary file 11 — (PNG 1934 kb) [file 13402_2022_713_Fig13_ESM.png]

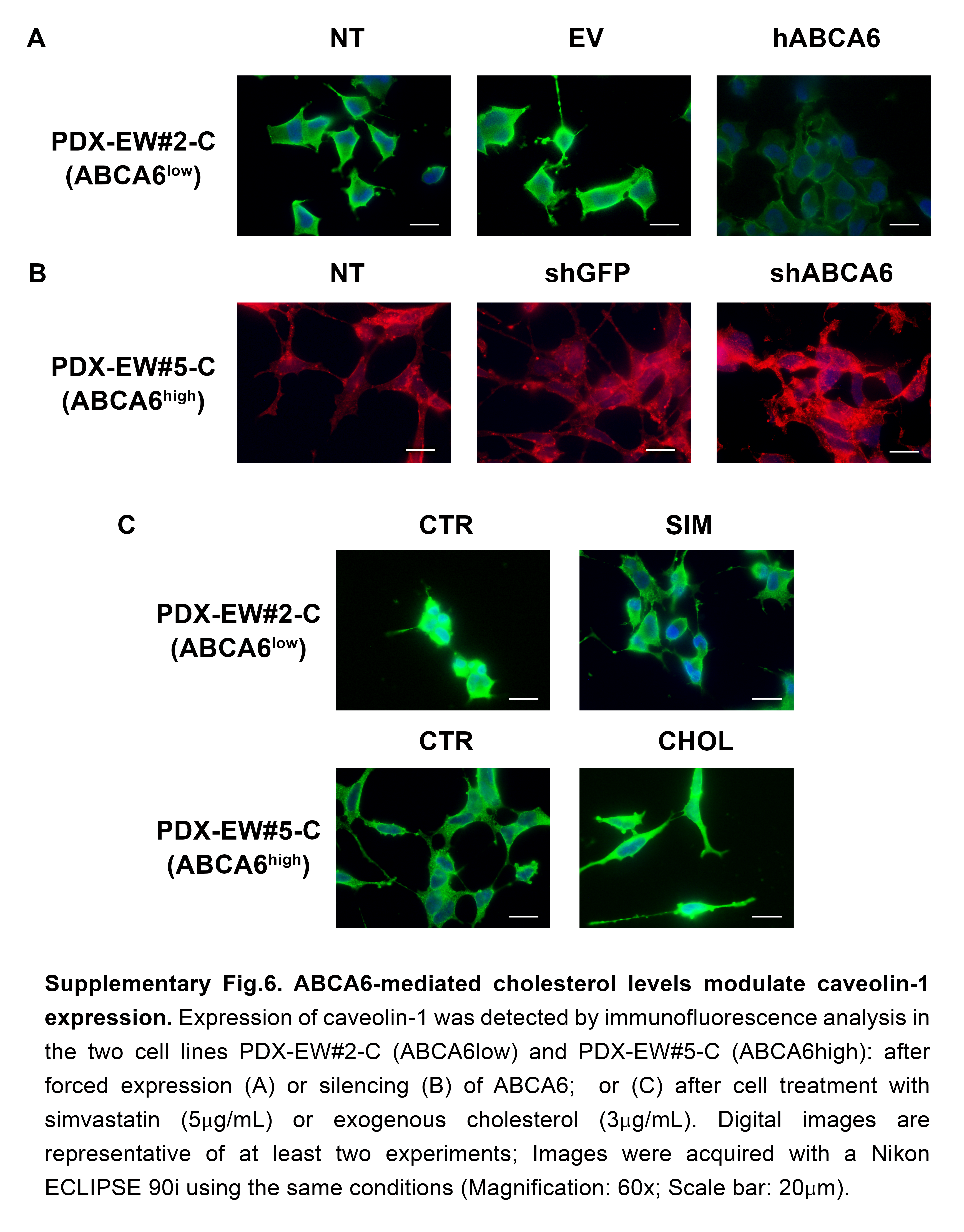

Supplement: Supplementary file 12 — High Resolution (TIF 32278 kb) [file 13402_2022_713_MOESM6_ESM.tif]
