# Supplementary material for: ABCA6 affects the malignancy of Ewing sarcoma cells via cholesterol-guided inhibition of the IGF1R/AKT/MDM2 axis
Source: Cell Oncol (Dordr). 2022 Sep 23;45(6):1237–51. doi: 10.1007/s13402-022-00713-5 (PMC9747862; doi:10.1007/s13402-022-00713-5)
Supplement: Supplementary file 13 — (DOCX 57.9 kb) [file 13402_2022_713_MOESM7_ESM.docx]

**Cellular Oncology**

**ABCA6 affects the malignancy of Ewing sarcoma cells via cholesterol-guided inhibition of the IGF1R/AKT/MDM2 axis**

Michela Pasello^1,§*^, Anna Maria Giudice^1,2,3,§^, Camilla Cristalli^1^, Maria Cristina Manara^1^, Caterina Mancarella^1^, Alessandro Parra^1^, Massimo Serra^1^, Giovanna Magagnoli^4^, Florencia Cidre-Aranaz^5,6^, Thomas G.P. Grünewald^5,6,7^, Carla Bini^8^ ,Pier-Luigi Lollini^3^, Alessandra Longhi^9^, Davide Maria Donati^10,11^ and Katia Scotlandi^1,2*^

*^1^Experimental Oncology Laboratory, IRCCS Istituto Ortopedico Rizzoli, Bologna, Italy; ^2^Alma Mater Institute on Healthy Planet - Alma Healthy Planet, University of Bologna, Bologna, Italy; ^3^Department of Experimental, Diagnostic and Specialty Medicine (DIMES), University of Bologna, Bologna, Italy; ^4^Department of Pathology, IRCCS Istituto Ortopedico Rizzoli, Bologna, Italy; ^5^ Division of Translational Pediatric Sarcoma Research, German Cancer Research Center (DKFZ), German Cancer Consortium (DKTK), Heidelberg, Germany; ^6^Hopp-Children’s Cancer Center (KiTZ), Heidelberg, Germany; ^7^Institute of Pathology, Heidelberg University Hospital, Heidelberg, Germany; ^8^Laboratory of Forensic Genetics, Department of Medical and Surgical Sciences, University of Bologna, Bologna, Italy; ^9^Osteoncologia, Sarcomi dell’osso e dei Tessuti Molli e Terapie Innovative, IRCCS Istituto Ortopedico Rizzoli, Bologna, Italy; ^10^Unit of 3rd Orthopaedic and Traumatologic Clinic Prevalently Oncologic, IRCCS Istituto Ortopedico Rizzoli, Bologna, Italy; ^11^Department of Biomedical and Neuromotor Sciences (DIBINEM), University of Bologna, Bologna, Italy.*

*^§^* These authors contributed equally to this work.

***To whom correspondence should be addressed:**

Dr. Katia Scotlandi [katia.scotlandi@ior.it](mailto:katia.scotlandi@ior.it).

Dr. Michela Pasello [michela.pasello@ior.it](mailto:michela.pasello@ior.it).

**Supplementary Methods**

**Survival analysis**

Microarray data of 166 primary Ewing sarcoma (EWS) tumors (accession codes: GSE63157 [[1](#_ENREF_1)], GSE34620 [[2](#_ENREF_2)], GSE12102 [[3](#_ENREF_3)], and GSE17618 [[4](#_ENREF_4)]), which had well-curated clinical annotations available, were downloaded from the National Center for Biotechnology Information (NCBI) Gene Expression Omnibus (GEO) and analyzed. These data were generated on Affymetrix HG-U133Plus2.0 or Affymetrix HuEx-1.0-st microarray chips as previously described [[5](#_ENREF_5)] and normalized separately by Robust Multiarray Average (RMA) using custom brainarray CDF files (v20) [[6-8](#_ENREF_6)]. Batch effects were removed using the ComBat algorithm (ComBat [[9](#_ENREF_9), [10](#_ENREF_10)]). Samples were stratified into groups according to their *ABCA6* expression status as ‘high’ and ‘low’ (cutoff 22^nd^ expression percentile). The cutoff was mathematically determined by testing all possible cutoffs between the 20^th^ and 80^th^ gene expression percentile, which yielded that the 22^nd^ expression percentile had the greatest discriminatory effect on the area under the curve in Kaplan-Meier survival analyses. Significance levels of differences between groups were calculated using the Mantel-Haenszel test. *p* < 0.05 was considered statistically significant.

**Sample processing for molecular analysis**

Total RNA from snap-frozen tissue samples and cell lines was isolated using TRIzol Reagent (Invitrogen) and quantity was assessed by NanoDrop analysis (Nanodrop ND-1000, ThermoFisher Scientific). To check whether the extracted RNA was representative of EWS, tissue sections from the same tumor samples were stained with hematoxylin- eosin, and a pathologist confirmed the presence of high tumor cell content (> 70%) in the respective areas from which the RNA sample was taken as previously reported [[11](#_ENREF_11), [12](#_ENREF_12)]. Tissues no representative of EWS were excluded.

Total RNA isolated from snap-frozen tissue samples or cell lines was reverse transcribed to cDNA using the High Capacity cDNA Reverse Transcription Kit (Applied Biosystems) according to the manufacturer’s instructions. Quantitative real-time PCR (RT-qPCR) was performed on ViiA7 (Life Technologies) using TaqMan PCR Master Mix (Life Technologies). The following predesigned TaqMan probes were used for the target gene of ABC transporters: *ABCA2* (Assay ID: Hs_00242232_m1), *ABCA6* (Assay ID: Hs_00365329_m1), *ABCA7* (Assay ID: Hs_00185303_m1), *ABCB1* (Assay ID: Hs_00184491_m1), *ABCB10* (Assay ID: Hs_00429240_m1), *ABCC1* (Assay ID: Hs_00219905_m1), *ABCC2* (Assay ID: Hs_00166123_m1), *ABCC4* (Assay ID: Hs_00195260_m1), *ABCC5* (Assay ID: Hs_00194701_m1), *ABCC11* (Assay ID: Hs_00261567_m1), *ABCE1* (Assay ID: Hs_00759267_m1), *ABCF1* (Assay ID: Hs_00153703_m1), *ABCF2* (Assay ID: Hs_00606493_m1), *ABCF3* (Assay ID: Hs_00217977_m1), *ABCG2* (Assay ID: Hs_00184979_m1) and *GAPDH* (Assay ID: Hs_99999905_m1). The expression levels of each *ABC* were normalized to that of *GAPDH* (housekeeping gene), and human derived hMSC mesenchymal stem cells were used as normal calibrators. The relative quantification (RQ) analysis was performed on the basis of the ΔΔCT method [[13](#_ENREF_13)].

**Cell exposure to simvastatin or exogenous cholesterol**

Cells were exposed to simvastatin (Sigma) or exogenous cholesterol (Sigma) as follows. PDX-EW#2-C and PDX-EW#5-C cells were seeded (5×10^5^ cells/well) in IMDM 10% FBS in 6-well plates and treated 24 h after seeding. For PDX-EW#2-C cells with ABCA6 forced expression or PDX-EW#5-C ABCA6-silenced cells, treatment was performed after 24 h of transfection. Cells were serum-starved for 24 h in IMDM 1% FBS and then exposed to simvastatin (1 μg/ml, 2.5 μg/ml, 5 μg/ml), exogenous cholesterol (0.3 μg/ml, 1 μg/ml, 3 μg/ml) or a combination of simvastatin plus cholesterol (5 μg/ml and 3 μg/ml, respectively) for 72 h. Harvested cells were used for evaluating *in vitro* parameters of malignancy, drug sensitivity, cholesterol detection and preparation of cell lysates for western blot.

***In vitro* parameters of malignancy**

Anchorage-independent growth was determined in 0.33% SeaPlaque Agarose (Lonza) with a 0.5% agarose underlay. To evaluate the effect of simvastatin or exogenous cholesterol on anchorage-independent growth, 1x10^4^ pretreated PDX-EW#2-C or PDX-EW#5-C cells were plated in semisolid medium supplemented with simvastatin (1 μg/ml, 2.5 μg/ml, 5 μg/ml) or exogenous cholesterol (0.3 μg/ml, 1 μg/ml, 3 μg/ml) respectively. Colonies were counted after 16 days.

The migration ability of EWS cells was assessed using Transwell chambers (Costar). To evaluate the effect of simvastatin or exogenous cholesterol on cellular migration, 1×10^5^ pretreated PDX-EW#2-C or PDX-EW#5-C cells were seeded in the upper compartment of the chamber in IMDM 10% FBS supplemented with simvastatin (1 μg/ml, 2.5 μg/ml, 5 μg/ml) or cholesterol (0.3 μg/ml, 1 μg/ml, 3 μg/ml); IMDM 10% FBS supplemented with the appropriate concentration of simvastatin or cholesterol was placed in the lower compartment. The migrated cells were fixed in absolute methanol, counterstained with Giemsa and counted.

Wound-healing assay was performed by seeding PDX-EW#2-C cells (1.5×10^6^/well) in IMDM 10% FBS in 60-mm-diameter petri dishes coated with fibronectin. Cells were allowed to grow until 100% confluence was achieved. A sterile 200-μl pipette tip was used to obtain the cell-free lane, and the medium was renewed with IMDM 10% FBS supplemented with simvastatin (5 μg/ml). Images were obtained at time 0 and after 24 h under an inverted microscope (Nikon Diaphot TMD).

***In vitro* drug sensitivity**

Sensitivity to conventional chemotherapeutics and simvastatin was assessed by 3-(4,5-dimethylthiazol-2-yl)-2,5-diphenyltetrazolium bromide (MTT) assay (TACS MTT Cell Proliferation Assays; Trevigen). A total of 4×10^4^ cells/well were seeded in 96-well plates in IMDM 10% FBS. After 24 h, the medium was changed with IMDM 10% FBS containing different concentrations of doxorubicin (1-100 ng/ml; Pfizer), vincristine (0.1-1000 ng/ml; Pfizer), ifosfamide (10-3000 ng/ml; Niomech - IIT GmbH), etoposide (10-3000 ng/ml; Sandoz) or simvastatin (1-250 μg/ml; Sigma). Sensitivity was assessed after 72 h of drug exposure according to the manufacturers’ protocol to estimate the percentage of growth inhibition when compared to controls (untreated cells). Sensitivity to different drugs was expressed as IC50 (drug concentration resulting in 50% inhibition of cell growth).

In combination experiments, 5×10^5^ PDX-EW#2-C or PDX-EW#5-C cells were seeded in IMDM 10% FBS in 6-well plates and treated 24 h after seeding. Cells were exposed to simvastatin (0 μg/ml, 5 μg/ml, 10 μg/ml) for 72 h and then treated for an additional 24 h with simvastatin alone (0 μg/ml, 5 μg/ml, 10 μg/ml) or in combination with doxorubicin. The following concentration ranges of doxorubicin were used: 0 ng/ml, 25 ng/ml, 50 ng/ml, 250 ng/ml for PDX-EW#2-C cells and 0 ng/ml, 5 ng/ml, 10 ng/ml, 25 ng/ml for PDX-EW#5-C cells. Accordingly, cells with ABCA6 forced expression or silencing were treated with the same experimental procedure after 24 h of transfection. Harvested cells were counted by Trypan blue vital cell count (Sigma). Sensitivity to different drug combinations was expressed as percentage of live cells.

To evaluate the reversal effect of exogenous cholesterol on the cytotoxic effect induced by simvastatin plus doxorubicin, 9×10^5^ PDX-EW#2-C cells/well were seeded in IMDM 10% FBS in 6-well plates and treated 24 h after seeding. Cells were exposed to simvastatin (0 μg/ml, 5 μg/ml) or simvastatin plus exogenous cholesterol (5 μg/ml and 3 μg/ml, respectively) for 72 h and then treated for an additional 3 h or 24 h with combinations of simvastatin (0 μg/ml, 5 μg/ml), exogenous cholesterol (0 μg/ml, 3 μg/ml) and doxorubicin (50 ng/ml). Harvested cells were counted by Trypan blue vital cell count (Sigma). Lysates for western blotting were collected. Sensitivity to different drug combinations was expressed as percentage of inhibition *vs* control (untreated cells).

**Mitochondrial membrane potential assay**

Changes in mitochondrial membrane potential were assessed by measuring 1,1′,3,3′-tetraethylbenzimidazolcarbocyanine iodide (JC-1; Sigma) red and green fluorescence intensity. PDX-EW#2-C and PDX-EW#5-C cells (5×10^5^/well) were seeded in 60-mm-diameter petri dishes; doxorubicin (5-50 ng/ml or 25-250 ng/ml, respectively) was added after 24 h. Otherwise, PDX-EW#2-C or PDX-EW#5-C cells (1x10^6^/well) were seeded in 6-well plates coated with fibronectin and after 24 h of transient transfection for forced expression or silencing of ABCA6 were treated with doxorubicin (250 ng/ml or 50 ng/ml, respectively, 24 h).

Harvested cells were exposed to JC-1 (5 μg/ml) for 15 min. The resulting fluorescence was measured by flow cytometry (Becton Dickinson), and the results are presented as percentages of cell green fluorescence. Additionally, sensitivity to doxorubicin was estimated in the cells under the same experimental conditions by Trypan blue vital cell count and expressed as IC50.

**Cholesterol detection and quantification**

Intracellular cholesterol was detected with filipin III staining. PDX-EW#2-C or PDX-EW#5-C cells (3x10^5^) were seeded on coverslips coated with fibronectin in IMDM 10% FBS. After 72 h, cells were rinsed with 1X PBS and fixed with 4% paraformaldehyde for 15 min at room temperature, incubated with glycine (1.5 mg/ml) for 10 min at room temperature and stained with filipin III (0.05 mg/ml in PBS/10% FBS; Sigma) for 2 h at room temperature. The fluorescence of the stained cells was detected using a Nikon A1 plus T1 confocal microscope with a Plan Apo VC 60x Oil DIC N2. Image analysis was performed using Nis Elements AR4.20.01 software (Nikon).

Intracellular and supernatant cholesterol quantification was obtained using the colorimetric Total Cholesterol Assay Kit (Cell Biolabs). Lipid extracts were obtained from 1x10^6^ cells using 200 μl of a chloroform: isopropanol: NP-40 (7:11:0.1, v:v:v) mixture and further processed according to the manufacturers’ protocol. Supernatants were pre-purified using Microcon centrifugal ultrafilters (Merck Millipore) and then cholesterol quantified.

**Western blotting**

Western blot experiments were performed according to standard protocols. Subconfluent cells were treated as described above and cell lysates were prepared using NP-40 1% lysis buffer (50 mM Tris–HCl pH 7.4, 150 mM NaCl, NP-40 1%, sodium deoxycholate 0.25%, 1 mM EGTA, 1 mM NaF, with protease and phosphatase inhibitors). Equal amounts of protein were analyzed by gel electrophoresis with a 4-15% separation gel (Mini-PROTEAN™ TGX Stain-Free™ Protein Gels; Biorad) or 10% gel and transferred to nitrocellulose membranes. Next, the membranes were incubated overnight with the following primary antibodies: anti-hABCA6 (Abcam, Cat#ab61093), anti-Cleaved Caspase-3 (Asp175; Cell Signaling Technology, Cat#9661), anti-PARP (Asp214; Cell Signaling Technology, Cat#9541), anti-phospho-AKT (Ser473; 736E11; Cell Signaling Technology, Cat#3787), anti-AKT (Cell Signaling Technology, Cat#9272), anti-phospho-mTOR (Ser2448; Cell Signaling Technology, Cat#2971S), anti-mTOR (Cell Signaling Technology, Cat#2972), anti-phospho-S6 Ribosomal Protein (Ser240/Ser244; Cell Signaling Technology, Cat#2215), anti-S6 Ribosomal Protein (54D2; Cell Signaling Technology, Cat#2317), anti-phospho-IGF-IRβ (Tyr1131, Cell Signaling Technology, Cat#3021), anti-IGF-IRβ (F-1; Santa Cruz Biotechnology, Cat#sc-390130), anti-phospho-MDM2 (Ser166; Cell Signaling Technology, Cat#3521), anti-MDM2 (Ab-2, 2A10; Calbiochem, Cat#OP115), anti-phospho-p53 (Ser15; Cell Signaling Technology, Cat#9284), anti-p53 (DO-1; SEROTEC, Cat#MCA1701) and anti-GAPDH (14C10; Cell Signaling Technology, Cat#2118). Anti-rabbit (GE Healthcare, Cat#NA934) or anti-mouse (GE Healthcare, Cat#NA931) antibodies conjugated to horseradish peroxidase were used as secondary antibodies. The proteins were visualized with an ECL western blotting Detection System (Euroclone).

**Immunofluorescence**

Cells were fixed with 4% paraformaldehyde, permeabilized with 0.15% Triton X-100 (Merck) in phosphate-buffered saline and incubated with the anti-caveolin-1 antibody (BD Transduction Lab, cat#610058 or cat#610059, dilution 1:100). A FITC-conjugated anti-mouse antibody (Bethyil, cat#A90-101F, dilution 1:100) or TRITC-conjugated rhodamine anti-rabbit antibody (Invitrogen, cat#31686, dilution 1:100) were used as secondary antibodies. Nuclei were counterstained with Hoechst 33258 (Merck). Images were acquired using a Nikon ECLIPSE 90i microscope and were then analyzed with NIS-Elements software (Nikon).

**References**

1. S. L. Volchenboum, J. Andrade, L. Huang, D. A. Barkauskas, M. Krailo, R. B. Womer, A. Ranft, J. Potratz, U. Dirksen, T. J. Triche, E. R. Lawlor, Gene Expression Profiling of Ewing Sarcoma Tumors Reveals the Prognostic Importance of Tumor-Stromal Interactions: A Report from the Children's Oncology Group. *J Pathol Clin Res* **1**, 83-94 (2015).

2. S. Postel-Vinay, A. S. Veron, F. Tirode, G. Pierron, S. Reynaud, H. Kovar, O. Oberlin, E. Lapouble, S. Ballet, C. Lucchesi, U. Kontny, A. Gonzalez-Neira, P. Picci, J. Alonso, A. Patino-Garcia, B. B. de Paillerets, K. Laud, C. Dina, P. Froguel, F. Clavel-Chapelon, F. Doz, J. Michon, S. J. Chanock, G. Thomas, D. G. Cox, O. Delattre, Common variants near TARDBP and EGR2 are associated with susceptibility to Ewing sarcoma. *Nat Genet* **44**, 323-327 (2012).

3. K. Scotlandi, D. Remondini, G. Castellani, M. C. Manara, F. Nardi, L. Cantiani, M. Francesconi, M. Mercuri, A. M. Caccuri, M. Serra, S. Knuutila, P. Picci, Overcoming resistance to conventional drugs in Ewing sarcoma and identification of molecular predictors of outcome. *J Clin Oncol* **27**, 2209-2216 (2009).

4. S. Savola, A. Klami, S. Myllykangas, C. Manara, K. Scotlandi, P. Picci, S. Knuutila, J. Vakkila, High Expression of Complement Component 5 (C5) at Tumor Site Associates with Superior Survival in Ewing's Sarcoma Family of Tumour Patients. *ISRN Oncol* **2011**, 168712 (2011).

5. M. C. Baldauf, M. F. Orth, M. Dallmayer, A. Marchetto, J. S. Gerke, R. A. Rubio, M. M. Kiran, J. Musa, M. M. L. Knott, S. Ohmura, J. Li, N. Akpolat, A. N. Akatli, O. Ozen, U. Dirksen, W. Hartmann, E. de Alava, D. Baumhoer, G. Sannino, T. Kirchner, T. G. P. Grunewald, Robust diagnosis of Ewing sarcoma by immunohistochemical detection of super-enhancer-driven EWSR1-ETS targets. *Oncotarget* **9**, 1587-1601 (2018).

6. M. C. Baldauf, J. S. Gerke, A. Kirschner, F. Blaeschke, M. Effenberger, K. Schober, R. A. Rubio, T. Kanaseki, M. M. Kiran, M. Dallmayer, J. Musa, N. Akpolat, A. N. Akatli, F. C. Rosman, O. Ozen, S. Sugita, T. Hasegawa, H. Sugimura, D. Baumhoer, M. M. L. Knott, G. Sannino, A. Marchetto, J. Li, D. H. Busch, T. Feuchtinger, S. Ohmura, M. F. Orth, U. Thiel, T. Kirchner, T. G. P. Grunewald, Systematic identification of cancer-specific MHC-binding peptides with RAVEN. *Oncoimmunology* **7**, e1481558 (2018).

7. M. Dai, P. Wang, A. D. Boyd, G. Kostov, B. Athey, E. G. Jones, W. E. Bunney, R. M. Myers, T. P. Speed, H. Akil, S. J. Watson, F. Meng, Evolving gene/transcript definitions significantly alter the interpretation of GeneChip data. *Nucleic Acids Res* **33**, e175 (2005).

8. R. A. Irizarry, B. Hobbs, F. Collin, Y. D. Beazer-Barclay, K. J. Antonellis, U. Scherf, T. P. Speed, Exploration, normalization, and summaries of high density oligonucleotide array probe level data. *Biostatistics* **4**, 249-264 (2003).

9. W. E. Johnson, C. Li, A. Rabinovic, Adjusting batch effects in microarray expression data using empirical Bayes methods. *Biostatistics* **8**, 118-127 (2007).

10. C. K. Stein, P. Qu, J. Epstein, A. Buros, A. Rosenthal, J. Crowley, G. Morgan, B. Barlogie, Removing batch effects from purified plasma cell gene expression microarrays with modified ComBat. *BMC Bioinformatics* **16**, 63 (2015).

11. F. Nakatani, M. Ferracin, M. C. Manara, S. Ventura, V. Del Monaco, S. Ferrari, M. Alberghini, A. Grilli, S. Knuutila, K. L. Schaefer, G. Mattia, M. Negrini, P. Picci, M. Serra, K. Scotlandi, miR-34a predicts survival of Ewing's sarcoma patients and directly influences cell chemo-sensitivity and malignancy. *J Pathol* **226**, 796-805 (2012).

12. M. T. Marino, A. Grilli, C. Baricordi, M. C. Manara, S. Ventura, R. S. Pinca, M. Bellenghi, M. Calvaruso, G. Mattia, D. Donati, C. Tripodo, P. Picci, S. Ferrari, K. Scotlandi, Prognostic significance of miR-34a in Ewing sarcoma is associated with cyclin D1 and ki-67 expression. *Ann Oncol* **25**, 2080-2086 (2014).

13. K. J. Livak, T. D. Schmittgen, Analysis of relative gene expression data using real-time quantitative PCR and the 2(-Delta Delta C(T)) Method. *Methods* **25**, 402-408 (2001).
