# Supplementary material for: ABCA6 affects the malignancy of Ewing sarcoma cells via cholesterol-guided inhibition of the IGF1R/AKT/MDM2 axis
Source: Cell Oncol (Dordr). 2022 Sep 23;45(6):1237–51. doi: 10.1007/s13402-022-00713-5 (PMC9747862; doi:10.1007/s13402-022-00713-5)
Supplement: Supplementary file 14 — (DOCX 31 kb) [file 13402_2022_713_MOESM8_ESM.docx]

**Supplementary Table 1.** **Clinicopathological features of patients affected by Ewing sarcoma.**

| **Characteristics** | **Training set**  **(25 cases)** | **Validation set**  **(78 cases)** |
| --- | --- | --- |
|  | *n* | *n* |
| **Gender** |  |  |
| Female | 9 (36%) | 21 (27%) |
| Male | 16 (64%) | 57 (73%) |
| **Age** |  |  |
| ≤ 14 years | 7 (28%) | 20 (26%) |
| > 14 years | 18 (72%) | 58 (74%) |
| **Location** |  |  |
| Extremity | 20 (80%) | 57 (73%) |
| Others | 5 (20%) | 21 (27%) |
| **LDH^a^** |  |  |
| Normal | 10 (66.7%) | 45 (79%) |
| High | 5 (33.3%) | 12 (21%) |
| **Surgery** |  |  |
| YES | 21 (84%) | 66 (85%) |
| NO | 4 (16%) | 12 (15%) |
| **Local Treatment^b^** |  |  |
| RxT | 4 (16%) | 11 (14%) |
| RxT + Surgery | 5 (20%) | 15 (20%) |
| Surgery | 16 (64%) | 51 (66%) |
| **Response to chemotherapy^c^** |  |  |
| Good | 5 (23.8%) | 12 (18%) |
| Poor | 16 (76.2%) | 54 (82%) |
| **RFS (Status)** |  |  |
| NED | 9 (36%) | 44 (56.4%) |
| REL | 16 (64%) | 34 (43.6%) |
| **OS (Status)** |  |  |
| Alive | 13 (52%) | 55 (70.5%) |
| Dead | 12 (48%) | 23 (29.5%) |

^a^data available for 15 patients for training set and for 57 patients for validation set. ^b^ data available for 77 patients for validation set; ^c^ data available for 21 patients for training set and for 66 patients for validation set. RFS, relapse-free survival (median follow-up: 21 months; range 4-328 months for 25 cases and median follow-up: 44 months; range 4-328 months for 78 cases); OS, overall-survival (median follow-up: 72 months; range 10-328 months for 25 cases and median follow-up: 61.5 months; range 4-328 months for 78 cases). LDH, lactate dehydrogenase; RxT, radiotherapy; NED, no evidence of disease; REL, relapsed.
