# Supplementary material for: ABCA6 affects the malignancy of Ewing sarcoma cells via cholesterol-guided inhibition of the IGF1R/AKT/MDM2 axis
Source: Cell Oncol (Dordr). 2022 Sep 23;45(6):1237–51. doi: 10.1007/s13402-022-00713-5 (PMC9747862; doi:10.1007/s13402-022-00713-5)
Supplement: Supplementary file 15 — (DOCX 25 kb) [file 13402_2022_713_MOESM9_ESM.docx]

**Supplementary Table 2. *ABCs* mRNA expression in 25 Ewing sarcoma clinical samples (training set).** Data are obtained by RT-qPCR and expressed as Log_2_RQ.

| **Sample code** | ***ABCA2*** | ***ABCA6*** | ***ABCA7*** | ***ABCB1*** | ***ABCB10*** | ***ABCC1*** | ***ABCC2*** | ***ABCC4*** | ***ABCC5*** | ***ABCC11*** | ***ABCE1*** | ***ABCF1*** | ***ABCF2*** | ***ABCF3*** | ***ABCG2*** |
| --- | --- | --- | --- | --- | --- | --- | --- | --- | --- | --- | --- | --- | --- | --- | --- |
| **L1** | 1.96391 | 8.24323 | 2.83944 | -3.18216 | 1.31396 | -0.40889 | 2.79321 | 0.54594 | 3.27593 | -2.13197 | 1.88504 | 2.69180 | 2.13231 | 3.68364 | 2.60484 |
| **L2** | 0.65951 | 0.84476 | 2.76060 | 3.42834 | -0.17929 | -1.66616 | -1.47440 | 0.59286 | 1.73982 | 0.48619 | 3.54617 | 1.33369 | -0.29346 | 1.74958 | -1.21352 |
| **L3** | -2.69864 | 7.77106 | -2.60102 | -5.89010 | -0.64031 | -3.07772 | -6.44352 | -3.86738 | 1.43972 | 7.36485 | -1.29310 | 0.28479 | -0.46340 | 0.96690 | 5.81796 |
| **L4** | 0.25100 | 8.11971 | 0.67877 | -0.99139 | 0.06730 | -1.11293 | -0.23902 | 0.45539 | 2.47207 | -3.08546 | 1.41896 | 1.18666 | 1.71549 | 2.00712 | 0.79353 |
| **L5** | 1.01723 | 6.84719 | -0.56283 | -2.23057 | 0.29037 | -0.90285 | -0.11769 | 2.30646 | 2.54958 | -3.30776 | 0.97336 | 1.15461 | 1.52358 | 2.08582 | 5.19365 |
| **L6** | -1.33582 | 0.72276 | -1.19714 | -8.10988 | -0.90242 | -2.23703 | -7.19802 | -1.76522 | -0.11177 | -5.23743 | -0.15435 | -0.10859 | 0.23175 | 0.39541 | -0.34280 |
| **L7** | -0.44102 | 4.27847 | 1.22174 | -2.79280 | -0.28633 | -1.40128 | 0.97434 | 0.10940 | 0.97169 | -3.03286 | -0.40738 | 0.79033 | 0.71531 | 1.36233 | 1.33294 |
| **L8** | -0.02138 | 8.14972 | 0.17396 | -5.21839 | 1.37554 | -1.33503 | 0.30943 | 1.01618 | 2.63057 | -4.95896 | 1.16673 | 1.48647 | 1.71352 | 2.46624 | 2.31062 |
| **L9** | 1.00031 | 9.38809 | 1.92845 | -0.46086 | 0.88340 | -0.23410 | 3.24630 | 0.51527 | 2.76492 | 0.56714 | 0.81873 | 2.08654 | 1.01536 | 1.92880 | 4.98283 |
| **L10** | -0.50597 | 6.52975 | -0.37499 | -4.64996 | 0.59246 | -1.07365 | -5.01004 | 1.69700 | 1.68565 | -3.04945 | 0.33673 | 0.79210 | 0.80391 | 1.49095 | 1.82570 |
| **L11** | 1.39705 | 6.75688 | 1.47985 | -2.94270 | 0.75279 | -1.02021 | -4.16403 | -0.99818 | 2.14235 | -2.20345 | 0.69079 | 1.59480 | 1.60169 | 2.28721 | 4.46912 |
| **L12** | -0.49987 | 2.79566 | -2.20708 | -7.04941 | -0.66813 | -2.45403 | 0.01836 | -0.79401 | 0.35987 | -4.46670 | 0.19123 | 0.17802 | -0.24601 | 1.63953 | 0.00817 |
| **L13** | 2.08390 | 7.05679 | 0.22657 | -2.81802 | 0.87933 | 0.58020 | 2.83206 | 2.68678 | 2.72220 | 2.96372 | 2.41497 | 2.90289 | 2.41486 | 3.25999 | 2.70653 |
| **L14** | -0.68269 | 5.64894 | -1.68563 | -4.60972 | 0.82810 | -1.36014 | -0.36828 | -1.68988 | 1.42062 | 0.09901 | -0.82038 | 0.04041 | 0.29772 | 1.08556 | 1.30866 |
| **L15** | -2.69778 | 1.85508 | -2.39610 | -2.10649 | -1.40561 | -1.82512 | -4.90689 | -2.01234 | -0.20850 | -2.94631 | 0.67356 | 1.10631 | 0.74020 | 0.31570 | -8.40346 |
| **L16** | -0.56999 | 3.76121 | -1.74941 | -3.53361 | -1.15087 | -2.64553 | -1.07224 | -1.28997 | 1.45621 | -4.26404 | -0.36469 | -0.31053 | 0.45565 | 0.71683 | 1.87568 |
| **L17** | -0.82065 | 5.39836 | -3.22533 | -7.42073 | -1.05125 | -3.52730 | -1.66131 | -1.26759 | -0.67492 | -1.62510 | -0.65835 | -0.68186 | 0.34201 | 0.48433 | -1.25776 |
| **L18** | -0.06838 | 8.19626 | -1.80053 | -2.96998 | 0.57371 | -2.49188 | -5.69086 | 1.89029 | 1.13380 | -3.73027 | 0.39324 | 0.66451 | 1.66194 | 2.11350 | 2.92751 |
| **L19** | -1.07858 | 3.96633 | -1.94606 | -5.25529 | 0.45871 | -1.35677 | 0.12264 | -0.80796 | 1.63757 | 0.15778 | -0.21758 | 0.25932 | 0.72370 | 0.79633 | 1.49734 |
| **L20** | 0.32426 | 4.02039 | 0.90667 | -3.68722 | 0.54031 | -1.01570 | -4.51788 | -0.61330 | 2.22408 | -2.55729 | 0.29090 | 1.26391 | 0.60255 | 2.27569 | 2.84991 |
| **L21** | -0.24226 | 6.77466 | -1.04933 | -4.12337 | 0.75135 | -0.46889 | -0.46179 | -1.21464 | 1.82615 | 1.12804 | 0.39876 | 0.93127 | 1.09931 | 1.79479 | 3.94191 |
| **L22** | 1.41111 | 8.17957 | -1.35985 | -3.41388 | -0.02026 | -4.00284 | -1.32505 | -3.05712 | 1.15365 | 1.19597 | 0.12785 | 0.09097 | 0.53830 | 2.29822 | 3.69101 |
| **L23** | 0.13532 | 2.35448 | 0.76389 | -1.11575 | 0.71111 | -0.52041 | 1.28040 | -1.19929 | 1.86321 | -2.24555 | 0.99579 | 1.68137 | 2.10552 | 2.35292 | 1.08311 |
| **L24** | -0.58221 | 7.79489 | -2.93871 | -8.33208 | -0.53543 | -5.44296 | -6.89498 | -1.07906 | 0.17990 | -4.93439 | -0.49992 | -0.33854 | 0.19956 | 1.23369 | 1.22428 |
| **L25** | -5.02361 | 4.51078 | -8.37576 | -8.53478 | -3.13060 | -5.77825 | -4.67465 | -5.12844 | -3.19072 | -2.71406 | -2.87304 | -2.40284 | -1.59255 | -2.84598 | -8.17122 |
|  |  |  |  |  |  |  |  |  |  |  |  |  |  |  |  |
| **Median value** | -0.24226 | 6.52975 | -1.04933 | -3.53361 | 0.29037 | -1.36014 | -1.07224 | -0.80796 | 1.63757 | -1.36014 | 0.33673 | 0.79210 | 0.72370 | 1.74958 | 1.82570 |
