# Supplementary material for: ABCA6 affects the malignancy of Ewing sarcoma cells via cholesterol-guided inhibition of the IGF1R/AKT/MDM2 axis
Source: Cell Oncol (Dordr). 2022 Sep 23;45(6):1237–51. doi: 10.1007/s13402-022-00713-5 (PMC9747862; doi:10.1007/s13402-022-00713-5)
Supplement: Supplementary file 16 — (DOCX 35 kb) [file 13402_2022_713_MOESM10_ESM.docx]

**Supplementary Table 3. *ABCs* mRNA expression in 78 Ewing sarcoma clinical samples (validation set).** Data are obtained by RT-qPCR and expressed as Log_2_RQ.

| **Sample code** | ***ABCA6*** | ***ABCA7*** |
| --- | --- | --- |
| **R1** | -1.507611275 | -0.339270592 |
| **R2** | 6.497124672 | 8.220442772 |
| **R3** | 1.826442719 | 2.319738388 |
| **R4** | 3.642972946 | 3.660527229 |
| **R5** | -1.199458122 | 0.713721275 |
| **R6** | 7.729712486 | 11.26064587 |
| **R7** | 2.022148132 | 2.175872803 |
| **R8** | 1.659479141 | 3.368994713 |
| **R9** | 1.577913284 | 3.088202477 |
| **R10** | 2.84256649 | 3.122968674 |
| **R11** | 2.094855309 | 3.067311287 |
| **R12** | 2.447990417 | 4.862133026 |
| **R13** | 1.981001854 | 4.863366127 |
| **R14** | 5.627989769 | 8.658312798 |
| **R15** | 4.733516693 | 3.080154419 |
| **R16** | 0.112660408 | 3.758026123 |
| **R17** | 3.060472488 | 2.932896614 |
| **R18** | 5.131557465 | 4.851867676 |
| **R19** | 3.259947777 | 3.859869003 |
| **R20** | 3.515983582 | 2.21743679 |
| **R21** | 4.452767372 | 14.22084618 |
| **R22** | 2.928666115 | 3.992429733 |
| **R23** | 0.732662201 | 2.905833244 |
| **R24** | 3.768382072 | 3.13365078 |
| **R25** | 3.290554047 | 3.602011681 |
| **R26** | 2.607255936 | 2.615347862 |
| **R27** | 3.732703209 | 4.757328987 |
| **R28** | 2.941117287 | 3.359233856 |
| **R29** | 2.617744446 | 2.135682106 |
| **R30** | 6.585599899 | 9.685587883 |
| **R31** | 9.04099369 | 9.978867531 |
| **R32** | 7.496455193 | 3.155432701 |
| **R33** | 5.011581421 | 6.656988144 |
| **R34** | 2.507112503 | 4.9013834 |
| **R35** | 11.65209293 | 17.16730595 |
| **R36** | -0.819925308 | 1.530692101 |
| **R37** | 6.81045025 | 10.68517807 |
| **R38** | -0.09040075 | 5.232456565 |
| **R39** | 5.21462025 | 7.653086565 |
| **R40** | 6.95492125 | 12.37747657 |
| **R41** | 2.93789475 | 9.635403065 |
| **R42** | 3.84444975 | 6.340559065 |
| **R43** | 2.28264025 | 6.511956565 |
| **R44** | 1.47448025 | 3.028922065 |
| **R45** | 4.31045325 | 7.537591065 |
| **R46** | 4.36126175 | 5.941629065 |
| **R47** | 2.09068575 | 7.930922565 |
| **R48** | 1.34689775 | 7.418602065 |
| **R49** | 4.19443675 | 3.731140565 |
| **R50** | -1.56428725 | 6.406074565 |
| **R51** | -0.51786425 | 3.868495065 |
| **R52** | 0.64677875 | 6.658655065 |
| **R53** | -2.47473325 | 9.000098065 |
| **R54** | 2.35866825 | 8.973942065 |
| **R55** | -1.238041182 | 4.961685065 |
| **R56** | 2.794957857 | 5.769312623 |
| **R57** | 4.286958437 | 7.04631305 |
| **R58** | 5.836955766 | 8.23531318 |
| **R59** | 3.83145688 | 1.726813081 |
| **R60** | -6.011542578 | 3.266314271 |
| **R61** | -2.347542067 | 5.728812936 |
| **R62** | 0.92945836 | 5.07081199 |
| **R63** | 2.721458177 | 3.632813218 |
| **R64** | 4.699457864 | 2.812811616 |
| **R65** | 1.490957002 | 4.093312981 |
| **R66** | 4.205958109 | 3.022813561 |
| **R67** | 1.923458795 | 6.182813409 |
| **R68** | 0.074959497 | 4.68731189 |
| **R69** | -1.318542738 | 4.267812493 |
| **R70** | 4.843456964 | 8.332313302 |
| **R71** | 1.443958025 | 3.640312913 |
| **R72** | -0.751042624 | 2.820311311 |
| **R73** | 0.278957109 | 2.633312943 |
| **R74** | -2.943541785 | 3.965313676 |
| **R75** | 1.45795701 | 5.025812867 |
| **R76** | -0.499542494 | 4.259312394 |
| **R77** | 4.708458643 | 4.490813973 |
| **R78** | 7.667957048 | 12.3038137 |
|  |  |  |
| **Median value** | 2.669601312 | 4.589062932 |
