# Supplementary material for: ABCA6 affects the malignancy of Ewing sarcoma cells via cholesterol-guided inhibition of the IGF1R/AKT/MDM2 axis
Source: Cell Oncol (Dordr). 2022 Sep 23;45(6):1237–51. doi: 10.1007/s13402-022-00713-5 (PMC9747862; doi:10.1007/s13402-022-00713-5)
Supplement: Supplementary file 17 — (DOCX 34 kb) [file 13402_2022_713_MOESM11_ESM.docx]

**Supplementary Table 4. Prognostic impact of clinicopathological features of *ABCA6 and ABCA7* in 78 patients with Ewing sarcoma (validation set).** Associations with prognosis were calculated by univariate analysis using the log-rank (Mantel-Cox) test.

|  |  | **Relapse-Free Survival** | |  | **Overall Survival** | |
| --- | --- | --- | --- | --- | --- | --- |
| **Characteristics** | ***N*** | **Events**  **(% RFS)** | **P Univariate** |  | **Events**  **(% OS)** | **P Univariate** |
| **Gender** |  |  | 0.526 |  |  | 0.723 |
| Female | 21 | 11 (41.0%) |  |  | 7 (60.0%) |  |
| Male | 57 | 23 (58.0%) |  |  | 16 (67.7%) |  |
| **Age** |  |  | 0.805 |  |  | 0.695 |
| ≤ 14 years | 20 | 10 (43.3%) |  |  | 6 (63.0%) |  |
| > 14 years | 58 | 24 (56.4%) |  |  | 17 (67.1%) |  |
| **Location** |  |  | **0.005** |  |  | 0.131 |
| Extremity | 57 | 31 (43.0%) |  |  | 20 (60.7%) |  |
| Others | 21 | 3 (84.7%) |  |  | 3 (82.6%) |  |
| **LDH^a^** |  |  | 0.354 |  |  | 0.089 |
| Normal | 45 | 20 (53.0%) |  |  | 12 (69.6%) |  |
| High | 12 | 7 (33.3%) |  |  | 6 (47.6%) |  |
| **Surgery** |  |  | 0.515 |  |  | 0.058 |
| YES | 66 | 28 (54.6%) |  |  | 17 (71.1%) |  |
| NO | 12 | 6 (46.3%) |  |  | 6 (34.7%) |  |
| **Local Treatment** |  |  | 0.754 |  |  | 0.069 |
| RxT | 11 | 6 (45.5%) |  |  | 6 (34.1%) |  |
| RxT + Surgery | 15 | 6 (59.3%) |  |  | 6 (57.8%) |  |
| Surgery | 51 | 22 (52.8%) |  |  | 11 (75.3%) |  |
| **Response to chemotherapy^b^** |  |  | **0.040** |  |  | **0.027** |
| Good | 12 | 2 (81.5%) |  |  | 0 |  |
| Poor | 54 | 26 (48.5%) |  |  | 17 (64.3%) |  |
| **RFS (Status)** |  |  |  |  |  |  |
| NED | 44 |  |  |  |  |  |
| REL | 34 |  |  |  |  |  |
| **OS (Status)** |  |  |  |  |  |  |
| Alive | 55 |  |  |  |  |  |
| Dead | 23 |  |  |  |  |  |
| ***ABCA6*** |  |  | **0.001** |  |  | **0.018** |
| Low | 39 | 24 (31.2%) |  |  | 16 (56.7%) |  |
| High | 39 | 10 (72.6%) |  |  | 7 (76.4%) |  |
| ***ABCA7*** |  |  | 0.365 |  |  | 0.382 |
| Low | 39 | 15 (61.3%) |  |  | 10 (67.0%) |  |
| High | 39 | 19 (46.7%) |  |  | 13 (63.1%) |  |

Results in bold are signiﬁcant at P < 0.05. ^a^data available for 57 patients. ^b^ data available for 77 patients; ^c^ data available for 66 patients. RFS, relapse-free survival median follow-up: 44 months; range 4-328 months; OS, overall-survival median follow-up: 61.5 months; range 4-328 months. LDH, lactate dehydrogenase; RxT, radiotherapy; NED, no evidence of disease; REL, relapsed.
