# Supplementary material for: ABCA6 affects the malignancy of Ewing sarcoma cells via cholesterol-guided inhibition of the IGF1R/AKT/MDM2 axis
Source: Cell Oncol (Dordr). 2022 Sep 23;45(6):1237–51. doi: 10.1007/s13402-022-00713-5 (PMC9747862; doi:10.1007/s13402-022-00713-5)
Supplement: Supplementary file 18 — (DOC 33 kb) [file 13402_2022_713_MOESM12_ESM.doc]

**Supplementary Table 5.** **Cox’s proportional hazards regression multivariate analysis.** Hazard ratio (HR) for variables associated with relapsed free survival (RFS) by univariate analysis in the dataset of 78 EWS patients estimated by Cox proportional-hazards regression multivariate analysis.

| **Variables associated with worse RFS** | **HR** | **95% CI** | ***P* value** |
| --- | --- | --- | --- |
| **Location:** extremity | 4.727 | 1.118-19.987 | **0.035** |
| **Response to chemoterapy:** POOR | 3.559 | 0.838-15.152 | 0.085 |
| **ABCA6 expression level:** low | 2.812 | 1.226-6.445 | **0.015** |
