# Supplementary material for: ABCA6 affects the malignancy of Ewing sarcoma cells via cholesterol-guided inhibition of the IGF1R/AKT/MDM2 axis
Source: Cell Oncol (Dordr). 2022 Sep 23;45(6):1237–51. doi: 10.1007/s13402-022-00713-5 (PMC9747862; doi:10.1007/s13402-022-00713-5)
Supplement: Supplementary file 19 — (DOCX 29 kb) [file 13402_2022_713_MOESM13_ESM.docx]

**Supplementary Table 6.** **Drugs sensitivity in PDX-derived cell lines.** Drugs sensitivity reported as IC50 values after 72 hours of treatment in PDX-derived cell lines.

| **PDX-derived**  **cell Lines** | **Doxorubicin**  **(ng/mL)** | **Vincristine**  **(ng/mL)** | **Etoposide**  **(ng/mL)** | **Ifosfamide**  **(ng/mL)** |
| --- | --- | --- | --- | --- |
| PDX-EW#2-C | 79.75 ± 10.65 | 5.67 ± 3.64 | 617.84 ± 36.87 | 1146.33 ± 135.68 |
| PDX-EW#5-C | 7.79 ± 0.22 | 1.68 ± 0.90 | 30.17 ± 4.77 | 311.08 ± 51.72 |
| *P value^a^* | *0.003* | *0.398* | *0.004* | *0.028* |

*^a^Student’s t-test.*
